# Discovery and Evaluation of Biomarkers for Triple-Negative Breast Cancer Subtypes Uncovers Patient Stratification and Targeted Therapeutic Strategies

**DOI:** 10.1158/0008-5472.CAN-24-2758

**Published:** 2026-02-11

**Authors:** Daniel Ortega-Álvarez, David Tébar-García, Marta Casado-Peláez, David Olivares-Osuna, Elena Castillo-Agea, Joan Balibrea-Rull, Rosa A. Barbella-Aponte, David Pérez-Parra, Eva Musulén, Cristina Guardia, Elena Vinuesa-Pitarch, Maria E. Sánchez-López, Fátima Postigo-Corrales, Ester Ballana, Anna Martínez-Cardús, Mireia Margelí, Manel Esteller, Pedro L. Fernández, Ginés Luengo-Gil, Elisabetta Mereu, Eva M. Galán-Moya, Verónica Rodilla

**Affiliations:** 1 Cancer Heterogeneity and Hierarchies Group, Josep Carreras Leukaemia Research Institute (IJC), Badalona, Spain.; 2 Cancer Pathophysiology and Therapy Lab, Institute of Biomedicine (IB-UCLM), Universidad de Castilla La Mancha, Albacete, Spain.; 3 Traslational Oncology Group UCLM-GAI Albacete, Universidad de Castilla-La Mancha, Servicio de Salud de Castilla-La Mancha, Albacete, Spain.; 4 Cellular Systems Genomics Group, Josep Carreras Leukaemia Research Institute (IJC), Badalona, Spain.; 5 Cancer Epigenetics Group, Josep Carreras Leukaemia Research Institute (IJC), Badalona, Spain.; 6 SITIO BioMedical Solutions S.A., Asunción, Paraguay.; 7 Department of Pathology, Complejo Hospitalario Universitario de Albacete, Albacete, Spain.; 8 Health Sciences Faculty, Universidad Católica de Murcia (UCAM), Guadalupe, Spain.; 9 Pathology and Clinical Analysis Department, Group of Molecular Pathology and Pharmacogenetics, Instituto Murciano de Investigación Biosanitaria (IMIB), Hospital Universitario Santa Lucía, Cartagena, Spain.; 10 Department of Pathology, Hospital Universitari General de Catalunya Grupo-QuirónSalud, Barcelona, Spain.; 11 Department of General Surgery, Hospital General Universitario de Albacete, Albacete, Spain.; 12 IrsiCaixa, Badalona, Spain.; 13 Centro de Investigación Biomédica en Red de Enfermedades Infecciosas, CIBERINFEC, Madrid, Spain.; 14 BARGO, CARE Program, Germans Trias i Pujol (IGTP), Universitat Autònoma de Barcelona, Badalona, Spain.; 15 Medical Oncology Department, Catalan Institute of Oncology-Badalona, Hospital Germans Trias i Pujol (HGTiP), Badalona, Spain.; 16 Centro de Investigacion Biomedica en Red Cancer (CIBERONC), Madrid, Spain.; 17 Institució Catalana de Recerca i Estudis Avançats (ICREA), Barcelona, Spain.; 18 Physiological Sciences Department, School of Medicine and Health Sciences, University of Barcelona (UB), Barcelona, Spain.; 19 Cancer Epigenetics Group, Sant Pau Research Institute (IRSantPau), Badalona, Spain.; 20 Department of Pathology, Hospital Germans Trias i Pujol, IGTP (Health Research Institute Germans Trias i Pujol), Universitat Autònoma de Barcelona, Badalona, Spain.; 21 Faculty of Nursing, Universidad de Castilla-La Mancha, Albacete, Spain.

## Abstract

Triple-negative breast cancer (TNBC) is the most heterogeneous and aggressive subtype of breast carcinoma, characterized by the absence of clinical biomarkers and the lack of targeted therapies. Despite numerous clinical trials, patient stratification remains suboptimal, limiting the identification of effective treatment strategies. In this study, we aimed to identify biomarkers exclusively expressed in the basal mammary epithelial compartment to refine TNBC subclassification. Computational analysis of single-cell RNA sequencing data defined a set of basal identity genes, which were subsequently validated by immunohistochemistry in two independent TNBC cohorts. This approach enabled the identification of a TNBC subgroup, termed true basal TNBC (tB-TNBC), which was associated with poorer prognosis. High-throughput screening of 3,200 FDA-approved compounds in breast cancer cell lines classified according to basal marker expression identified dasatinib as a promising candidate with selective activity against tB-TNBC models. In tB-TNBC patient-derived xenograft models, dasatinib treatment effectively suppressed tumor growth. Furthermore, *TAGLN* emerged as a strong predictive biomarker of dasatinib response, with functional studies confirming its role in modulating drug sensitivity. Altogether, these findings support the clinical utility of basal markers for TNBC stratification and highlight a targeted treatment opportunity for patients with tB-TNBC.

**Significance:**

Basal biomarkers enable subclassification of triple-negative breast cancer and reveal a specific tumor subgroup sensitive to dasatinib, providing an effective stratification and treatment strategy for patients.

## Graphical Abstract

**Figure:**
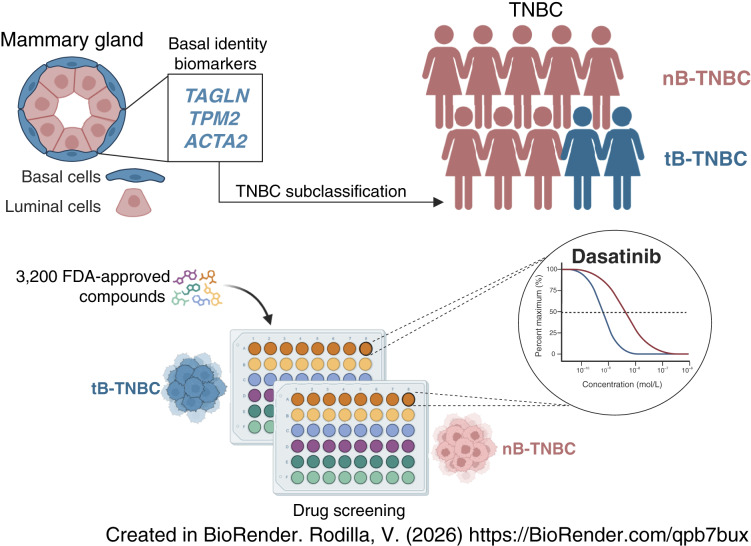

## Introduction

Breast carcinomas are clinically classified based on the histologic presence of hormone receptors, such as estrogen receptor α (ERα) and progesterone receptor (PR), as well as the overexpression of human epidermal growth factor receptor 2 (HER2; ref. 1). Tumors expressing ERα and PR are categorized as hormone receptor (HR)-positive, whereas those with elevated HER2 levels are classified as HER2-enriched (1). In contrast, tumors lacking these clinical biomarkers are diagnosed as triple-negative breast cancer (TNBC), which accounts for approximately 15% of all breast cancer cases. TNBC is associated with a poor prognosis due to its aggressive nature, its higher prevalence among younger women (2), and the absence of targeted therapy options, leaving chemotherapy as the standard treatment (2).

Identifying new biomarkers and molecular targets for TNBC has been a daunting challenge because of its biological heterogeneity, which is evident in the variability in prognosis, pathologic characteristics, treatment responses, and gene expression profiles (3). The discovery of HER2 as a breast cancer marker exemplifies how novel biomarkers can first recognize patients with different outcomes and, second, aid in developing new targeted therapies, such as monoclonal antibodies, small molecules, or chimeric antigen receptor T cells. Therefore, the quest for molecular markers that can target specific tumor types has been a focal point of research. To understand breast tumor heterogeneity, several transcriptomic panels, such as PAM50, which classifies breast cancer into luminal A/B, HER2-enriched, basal-like, and normal-like subgroups (4, 5), and the 70-gene MammaPrint microarray assay, which has prognostic significance in ERα-positive and ERα-negative early-stage node-negative breast cancer (6), have been developed over the last decades. Specifically, for TNBC, Lehmann’s classification identified up to four distinct subgroups (basal-like 1 and 2, mesenchymal-like, and luminal androgen receptor; ref. 7). These transcriptomic-based studies have clearly demonstrated that tumor heterogeneity has cemented the concept of breast cancer as a collection of different pathologies, each with varied clinical outcomes, rather than a singular disease (8, 9). Crucially, the clinical diagnosis of TNBC could be greatly enhanced through the discovery of novel molecular biomarkers that enable histologic stratification, thereby contributing to the development of new targeted therapies and significantly improving the prognosis of these patients.

The epithelial compartment of the mammary gland is composed of basal cells (BaC), which line the outer layer of the ducts and are known for their contractile ability, and luminal cells (LC), which line the central lumen. LCs are subdivided into ERα-expressing cells (ERα^pos^ cells) and hormone-sensing cells that can induce proliferation of the surrounding luminal ERα-negative cells (ERα^neg^ cells), often referred to as hormone-responsive or alveolar cells (10). Lineage-tracing experiments in healthy murine mammary glands have shed light on the cellular hierarchy within this tissue. These studies have shown that, in adult mice, different epithelial cell populations of the mammary gland are self-maintained by specific unipotent cells, indicating that these epithelial types contribute independently to mammary gland homeostasis by preserving their cellular identities into adulthood (11–13). Recent single-cell RNA sequencing (scRNA-seq) studies of the human mammary gland have revealed similar epithelial cellular populations (14, 15). Notably, the molecular markers ERα and PR, currently used in breast cancer diagnosis, only positively identify luminal ERα^pos^ cells, whereas the other two epithelial cell types, luminal ERα^neg^ and BaCs, remain histologically indistinct in clinical practice. Given that TNBC subtype consists of negative cells for these tested markers, it is intriguing to consider that defining new molecular markers that can distinguish each mammary epithelial cell type may significantly enhance our understanding of breast cancer heterogeneity. This allowed us to classify tumors with distinct cellular identities at the histopathologic level and characterize differences in their metastatic capacity and/or drug resistance, ultimately addressing two unmet clinical needs.

In this study, we characterized novel molecular basal-associated markers [smooth muscle cell actin (SMA), transgelin (TAGL), and β-tropomyosin (TPM2)] that play crucial roles in driving the malignancy of a specific subset of TNBCs. Remarkably, the expression of these biomarkers not only correlated with the aggressive nature of this breast cancer subtype but also significantly influenced their sensitivity to clinically approved drugs, such as dasatinib. Dasatinib, a multi-kinase inhibitor traditionally used in the treatment of certain hematologic cancers (16, 17), emerges as a promising therapeutic option for this specific subset of TNBC. Here, we propose using basal-associated markers to stratify patients with TNBC, thereby identifying those who would benefit from dasatinib treatment. This approach represents a new direction in the personalized treatment of TNBC, offering hope for improved outcomes in cancer subtypes that have historically been challenging to manage.

## Materials and Methods

### scRNA-seq analysis

We initially preprocessed each scRNA-seq dataset independently, including murine healthy mammary glands (GSE109711, GSE164017, and GSE148791) and healthy human breast tissue (GSE161529, GSE180878, and GSE113197). For two murine datasets, GSE109711 (18) and GSE164017 (19) datasets, raw count matrices were available. The GSE148791 (20) dataset was provided as a Seurat object, and only wild-type basal and luminal cell populations were retained for downstream analysis. The human raw count matrices were processed similarly, with the following samples retained from each dataset: three samples (RM-A, RM-B, and RM-C) from the Gray dataset (21); four samples (Ind4, Ind5, Ind6, and Ind7) from the Nguyen dataset (14); and 11 samples (N-1469-Epi, N-N1105-Epi, N-N280-Epi, N-0064-Epi, N-0093-Epi, N-0123-Epi, N-0230.16-Epi, N-0275-Epi, N-0342-Epi, N-0372-Epi, and N-0408-Epi) from the Pal dataset (15). All analyses were conducted using Seurat (v4.0.4 and v5.1.0 for murine and human analyses, respectively; refs. 22, 23) in R (4.4.1 and 4.0.3 for murine and human analyses, respectively). Seurat objects were created for each dataset and subsequently merged by sample for downstream analysis. Quality control (QC) was conducted to ensure the inclusion of high-quality cells across all datasets. Mitochondrial gene expression was quantified using *PercentageFeatureSet* function, and cells with mitochondrial content exceeding dataset-specific thresholds (ranging from 8% to 25%) were excluded. Cells with fewer than around 500 detected genes were excluded as low-quality cells, except for the murine Wuidart dataset (18), in which a higher threshold of 2,500 detected genes was applied due to deeper sequencing. No additional QC was required for the murine Centonze (20) and Gray (21) datasets, as they were already prefiltered. In the human datasets, we filtered out potential artificial doublets generated during library construction using two independent doublet detection methods: DoubletFinder (version 2.0.4; ref. 24) and scDblFinder (version 1.20.0; ref. 25). Cells classified as doublets by both methods were excluded for downstream analysis. Normalization was performed for all datasets (NormalizeData function: “LogNormalize” method and a scale factor 1e4). Dimensionality reduction was performed via principal component analysis (RunPCA function) on the scaled expression (ScaleData function) of the top highly variable genes [FindVariableFeatures function (variance-stabilizing transformation method). The top 20 PCs were used to generate Uniform Manifold Approximation and Projection (UMAP) embedding (RunUMAP function) and cluster the cells (FindClusters function) in a 20–nearest neighbor graph (FindNeighbors function).

Cluster annotations were performed based on differentially expressed genes (DEG) and were manually identified using canonical markers. For the murine datasets, three main populations were identified using key marker genes: ER-negative (*Elf5* and *Krt8*), ER-positive (*Prlr* and *Krt8*), and basal (*Krt5* and *Acta2*). The human datasets contained a more diverse array of cell types, which were annotated according to cell type–specific gene signatures, as described in Gray and colleagues (21). After processing each dataset individually, murine and human datasets were integrated separately. Batch correction was performed using Seurat’s canonical correlation analysis integration pipeline for the murine datasets and the Harmony algorithm (26) for the human datasets, implemented via Seurat’s IntegrateLayers function with the HarmonyIntegration method. For human data integration, the merged Seurat object was first split by sample, followed by normalization and independent identification of variable features within each batch. The harmony embeddings were then used to compute the top 20 PCs, which were subsequently used to construct UMAP embeddings and perform clustering using a 20–nearest neighbor graph. Cluster annotations were refined based on canonical markers in the integrated objects. Gene signature scoring was performed using UCell (27) AddModuleScore_UCell function. Differential expression analysis was conducted using the Seurat’s FindAllMarkers function to identify DEGs between clusters or annotated populations.

### Human paraffin-embedded tissue samples

In this study, three distinct cohorts of breast cancer samples were analyzed. The first cohort (discovery cohort) comprised 80 samples, including 26 TNBC cases from Hospital Germans Trias i Pujol and the General University Hospital of Albacete and 54 HR-positive breast cancer samples encompassing low-grade ductal carcinoma *in situ* (LGDCIS; *n* = 10), DCIS (DCIS; *n* = 23), and invasive ductal carcinoma (IDC; *n* = 21), collected from Hospital Germans Trias i Pujol, Albacete University Hospital, and Hospital Universitario Santa Lucía. These samples represent independent, nonpaired cases from different patients across disease stages. The second and third cohorts (validation cohorts) consisted of a commercial TNBC tissue array (BR1301a; *n* = 120), with one core per case, and a TNBC tissue microarray (TMA) comprising 123 cases from Hospital Universitario Santa Lucía. Detailed clinical and pathologic data were available for these samples, including tumor grade, tumor–node–metastasis classification, age at diagnosis, clinical stage (AJCC eighth edition), and immunohistochemistry (IHC) results for ERα, PR, and HER2 (Supplementary Tables S1 and S2).

### Ethical compliance

This study was conducted in accordance with the Declaration of Helsinki and all applicable national regulations governing biomedical research involving human subjects. Clinical patient samples were collected with approval from the Ethical Committee of Germans Trias i Pujol Hospital and General University Hospital of Albacete (CEIC #PI-21-230). Written informed consent was obtained from all participants prior to their inclusion in the study. All animal procedures in this research were approved by the Ethical Committee for Animal Research of the Comparative Medicine and Bioimage Centre of Catalunya and were performed in accordance with the regulations of the Departament de Medi Ambient i Habitatge of the Generalitat de Catalunya (Catalonia Government). Euthanasia was performed in all experimental procedures at the experimental endpoint or if the animals’ health was otherwise compromised.

### IHC analysis

Formalin-fixed and paraffin-embedded samples, serial sectioned at 4 μm, were dewaxed using a graded series of ethanol. Antigen retrieval was performed by boiling the sections for 20 minutes in either a citrate buffer solution (C9999, Sigma-Aldrich) or Tris-HCl buffer solution (T6455, Sigma-Aldrich). To block endogenous peroxidase activity, sections were incubated for 10 minutes in 3% hydrogen peroxide solution. Permeabilization and blocking were achieved using blocking buffer containing 0.3% Triton X-100, 2% BSA, and 5% FBS. The sections were then incubated overnight at 4°C with primary antibodies diluted in blocking buffer at the concentrations indicated in Supplementary Table S3. This was followed by 1-hour room temperature incubation with secondary antibodies (Supplementary Table S4). Sections were developed using the Signal Stain DAB Substrate Kit (8059S, Cell Signaling Technology) according to the manufacturer’s instructions and counterstained with hematoxylin. Mounting was performed using DPX mounting solution for histology (06522, MERCK). Whole tissue images were captured using Panoramic SCAN II and analyzed using the *QuPath* software. Tumor areas were delineated by a pathologist in images using the Brightfield (H-DAB) format, and a positive cell detection algorithm was applied to identify positive and negative cells and quantify the percentage of positive cells within the selected areas.

### Analysis of transcriptomic data from The Cancer Genome Atlas cohort

RNA-seq expression data for The Cancer Genome Atlas (TCGA) cohort were downloaded from FireBrowse (http://firebrowse.org/?cohort=BRCA&download_dialog=true), which provides processed datasets from the TCGA project (28). Only samples corresponding to patients with TNBC were selected for analysis. Expression levels of *ACTA2*, *TAGLN*, and *TPM2* were extracted and mean-centered by gene. A heatmap was generated using hierarchical clustering of the samples based on pairwise Euclidean distances, highlighting patterns of expression similarity across the three genes.

### Immune checkpoint and immunotherapy response analysis

Publicly available transcriptomic data from the phase II I-SPY2 clinical trial (GSE173839) were analyzed to evaluate the association between true basal (tB) marker expression and response to immunotherapy. In this trial, patients with TNBC were treated in the neoadjuvant setting with paclitaxel either alone or in combination with durvalumab (anti–PD-L1) and olaparib. Normalized gene expression data were retrieved and stratified by pathologic response status: patients who achieved pathologic complete response (pCR) and those who did not (non-pCR). Expression levels of *TAGLN*, *ACTA2*, and *TPM2* were extracted and compared between groups using unpaired *t* tests. In parallel, a cohort of 176 TNBC samples (28) was analyzed to assess the expression of selected immune checkpoint–related genes, including *PDCD1* (PD-1), *CD274* (PD-L1), *CTLA4*, *TIGIT*, and *PVR* (29). Gene expression data were normalized by *z*-score and visualized in a heatmap. Samples were annotated according to PAM50 and Lehmann subtypes, as well as their tB-TNBC classification status, to explore potential subtype-specific immune expression patterns.

### Survival analysis using public datasets

To evaluate the prognostic significance of the identified basal markers, two complementary survival analysis tools were used: the Kaplan–Meier (KM) Plotter and bc-GenExMiner. KM Plotter (https://kmplot.com) was used to assess the combined prognostic value of *ACTA2*, *TAGLN*, and *TPM2* in TNBC. The analysis was performed on the mRNA (gene chip) dataset, and the endpoints analyzed included recurrence-free survival (RFS) and overall survival (OS). For each gene, the following probe sets were used: *TAGLN* (“1555724_s_at” and “205547_s_at”), *TPM2* (“212654_at,” “227397_at,” and “204083_s_at”), and *ACTA2* (“215787_at” and “200974_at”). The average expression of the three genes was computed, and patients were stratified into high or low expression groups based on the upper and lower quartiles. Patients in the intermediate quartile were excluded to strengthen the contrast between groups. For RFS and OS, the analysis was restricted to patients with TNBC (defined by ER−, PR−, and HER2-negative status). No additional filters were applied for tumor grade, nodal status, or molecular subtype. Hazard ratios (HR) with 95% confidence intervals and log-rank *P* values were calculated, and patient numbers at risk were indicated below each KM curve. In parallel, individual prognostic relevance of *ACTA2*, *TAGLN*, and *TPM2* was assessed using bc-GenExMiner v4.10 (http://bcgenex.centregauducheau.fr). This online tool integrates breast cancer gene expression datasets with annotated clinical information, allowing survival analysis based on distant metastasis-free survival, disease-free survival, and OS. Patients were grouped into “high” or “low” expression cohorts based on automatic mean split, and KM curves were generated. Log-rank *P* values and HRs were provided by the platform.

### Cell line stratification for *in vitro* studies

Transcriptomic profiles of breast cancer cell lines were obtained from the publicly available dataset (DepMap) and processed using the DESeq2-normalized counts. Expression values for *ACTA2*, *TAGLN*, and *TPM2* were extracted, scaled, and combined to generate a composite tB-marker score for each cell line. Hierarchical clustering was performed using Euclidean distance and complete linkage on the scaled expression matrix to classify cell lines into tB- and nonbasal (nB)-TNBC groups. Hs578T and BT-549 cells, which showed the highest combined expression of the three tB-markers, were selected as representative tB-TNBC models. Conversely, MDA-MB-468 and HCC70 cells displayed consistently low expression of all three markers and were selected as representative nB-TNBC models. These classifications guided the selection of cell lines for subsequent *in vitro* functional assays and drug screening studies.

### Cell culture

TNBC cell lines HS578T (ACC781, DSMZ; RRID: CVCL_0332) and BT-549 (HTB-122, ATCC; RRID: CVCL_1092) and HR-positive cell line MCF7 (ACC115, DSMZ; RRID: CVCL_0031) were cultured in DMEM/F-12 with GlutaMAXTM Supplement medium (31331093, GibcoTM) supplemented with 10% FBS, at 37°C and 5% CO_2_ atmosphere. TNBC cell lines MDA-MB-468 (CRM-339 HTB-26, ATCC; RRID: CVCL_0419) and HCC70 (CRL-2315, ATCC; RRID: CVCL_1270), along with HR-positive cell line and T-47D (DSMZ, ACC739; RRID: CVCL_0553), were cultured with Advanced RPMI 1640 medium (12633020, GibcoTM) supplemented with 10% FBS, at 37°C and 5% CO_2_ atmosphere. For CRISPR/Cas9 knockout (KO) transfection, Hs578T and BT-549 cells were cotransfected with a ribonucleoprotein (RNP) complex comprising Cas9 and guide RNA (gRNA), according to the manufacturer’s instructions (CMAX00015, Invitrogen). crRNA and tracrRNA (1072534, IDT) were mixed at equimolar concentrations, heated at 95°C for 5 minutes, and allowed to cool at room temperature. To assemble the RNP complex, Cas9-GFP enzyme (10008161, IDT) was prepared at 1 μmol/L in Opti-MEM Reduced Serum Medium (31985047, Gibco). Each gRNA was then combined with the Cas9-GFP in Cas9 PLUS Reagent and Opti-MEM. The RNP complex was subsequently mixed with CRISPRMAX transfection reagent at room temperature for 20 minutes. After 24 hours at 37°C, transfected cells were plated in 96-well dishes for cellular subcloning. For PDGFRB overexpression, lentiviral particles were generated using the PDGFRB Human Tagged Lenti ORF Clone (RC206377L4, OriGene) plasmid and used to transduce target cells. Following infection, cells were selected with puromycin (5 μg/mL) for 10 days and subsequently maintained in culture under standard conditions. Cell lines were regularly tested for *Mycoplasma* and were authenticated using short tandem repeat profiling yearly.

### High-throughput drug screening using FDA-approved compound library

To identify compounds with selective cytotoxicity against tB-TNBC cells, a high-throughput screening was performed using a library of 3,200 FDA-approved drugs (Selleckchem, L1300). Cells were seeded in 96-well plates at a density of 5,000 cells per well and incubated 24 hours before treatment. Each compound was added at a final concentration of 10 μmol/L and incubated for 72 hours at 37°C in a humidified CO_2_ incubator. After treatment, cells were fixed with 4% formaldehyde for 10 minutes and stained with crystal violet. Plates were washed and air-dried, and the dye was solubilized with acetic acid. Absorbance was measured at 590 nm using an AGILENT Biotek Synergy H1 reader. For data analysis, viability values from the four cell lines were averaged by group to obtain mean viabilities for tB-TNBC and nB-TNBC. Compounds were considered hits if they reduced the mean viability of the tB-TNBC group by more than 80% while maintaining a mean viability above 20% in the nB-TNBC group, thereby identifying compounds with selective cytotoxicity toward tB-TNBC cells.

### Immunofluorescence

Hs578T and BT-549 cells were fixed with a 4% paraformaldehyde (PFA) solution for 10 minutes at room temperature. Permeabilization and blocking were performed using a blocking solution containing 0.3% Triton X-100, 2% BSA, and 5% FBS for 1 hour at room temperature. The cells were then incubated for 1 hour at room temperature with primary antibodies diluted in blocking solution at the concentrations indicated in Supplementary Table S5. This was followed by incubation with the secondary antibodies (Supplementary Table S4) for 1 hour at room temperature. Mounting and DNA/nuclear staining were subsequently performed using Fluoroshield mounting medium with DAPI. Images were captured using an EVOS M500 microscope and processed using Fiji–ImageJ software (RRID: SCR_002285).

### Western blot

For protein extraction, cells were pelleted and washed with cold PBS before being lysed in RIPA buffer (89900, Thermo Scientific) supplemented with 10 μL/mL Halt Protease and Phosphatase Inhibitor Cocktail EDTA-Free (78440, Thermo Scientific). Following centrifugation at 14,000 × *g* for 15 minutes, the supernatant was collected to quantify the protein concentration using a bicinchoninic acid Pierce protein assay. Prior to electrophoresis, samples were heated at 95°C for 5 minutes. Running Buffer 1X was prepared by diluting MES SDS Running Buffer 20X (J62138, Thermo Scientific Chemicals). A precast gel was placed in the tank and filled with Running Buffer 1X. The samples were loaded onto the gel and electrophoresed at a constant voltage of 200 V. Protein transfer was performed using an iBlot Gel Transfer Device (IB21001, Invitrogen), according to the manufacturer’s instructions. The membranes were blocked in a buffer containing 5% milk and 0.1% Tween-20 in PBS for 1 hour at room temperature. After blocking, the membranes were incubated overnight at 4°C with primary antibodies (Supplementary Table S5) diluted in 5% BSA and 0.1% Tween-20 in PBS with shaking. The next day, the membranes were incubated with secondary antibodies (Supplementary Table S4) for 1 hour at room temperature. ECL solution (32109, Thermo Scientific) was used for protein detection, and images were captured using a Vilber Smart Imaging Fusion FX developer.

### Proliferation assay

To conduct the CellTiter 96 Non-Radioactive Cell Proliferation Assay (MTT; G4000, Promega), 5,000 cells were seeded in 100 μL of DMEM/F-12 with GlutaMAX Supplement medium containing 10% FBS in each well of a 96-well plate at designated time points (24, 48, and 72 hours). The plates were then incubated at 37°C in a humidified CO_2_ incubator. At each time point, the dye solution was added to each well and the cells were incubated at 37°C for 4 hours, shielded from light. Following this incubation, the stop solution was added, and the cells were further incubated for 1 hour at 37°C. The absorbance was subsequently measured at 570 nm using an AGILENT Biotek Synergy H1 multiplate reader.

### Migration assay

To assess cell migration, 10,000 cells were seeded in a 12-well insert (9318002, CellQART) with an 8 μm pore size containing 350 μL of FBS-free medium. Chemoattractant-containing medium, consisting of DMEM/F-12 with GlutaMAX supplement and 10% FBS, was added to the bottom well. The cells were then incubated for 48 hours at 37°C in a humidified CO_2_ incubator. After incubation, the medium was removed from the insert, and nonmigrated cells were gently swabbed from the top of each insert. The inserts were fixed with 4% PFA for 15 minutes and stained with crystal violet (C0775, Sigma-Aldrich) for 10 minutes. After drying, the stain was solubilized in acetic acid solution (5330010050, Supelco), and the absorbance was read at 590 nm using an AGILENT Biotek Synergy H1 multiplate reader.

### RNA extraction, sequencing, and analysis

Total RNA was extracted using the NZY miRNA Isolation & RNA Clean-up Kit (MB13402, NZYtech; RRID: SCR_016772) following the manufacturer’s protocol. The purity and concentration of the RNA from each sample were assessed using an Agilent TapeStation using the RNA ScreenTape Analysis Kit. The samples sent for sequencing had RIN values above 8. mRNA enrichment was subsequently performed using poly(A)-tail–connected magnetic beads and oligos. This was followed by double-stranded DNA synthesis and polymerase chain reaction (PCR) amplification using specific primers. The PCR products were subjected to thermal denaturation to produce single-stranded DNA, which was cyclized into a circular DNA library using bridge primers. Sequencing was conducted using the DNBSEQ platform (BGI Genomics, Co.; RRID: SCR_015025). Raw sequencing data were preprocessed using *SOAPnuke* software (BGI Genomics, Co.). This involved removing reads with adapter contamination, more than 5% N content, and low-quality reads (in which more than 20% of the bases had a quality score below 15). The resulting “clean reads” were saved in FASTQ format. RNA-seq data were analyzed using the Galaxy workbench platform (RRID: SCR_006281; ref. 30), adhering to specific recommendations (31). Quality control and trimming of the reads were performed using MultiQC (RRID: SCR_014982; ref. 32) and Cutadpt (33). The reads were then mapped to the reference genome (Hg38, human genome build 38) using STAR (RRID: SCR_004463; ref. 34). From the mapped sequences, the number of reads per annotated gene was counted using *featureCounts* (RRID: SCR_012919; ref. 35). Subsequently, DESeq2 (RRID: SCR_015687; ref. 36) was used to normalize the read counts and identify DEGs. Functional enrichment analysis of DEGs was performed using gene set enrichment analysis (GSEA; RRID: SCR_003199; ref. 37).

### *In vitro* drug response assay

A total of 5,000 cells were seeded in 96-well plates and incubated at 37°C in a humidified CO_2_ incubator. After 24 hours, cells were treated with serial dilutions of dasatinib starting at 1 mmol/L (Selleckchem, S1021) and incubated for an additional 72 hours at 37°C. The cells were fixed with 0.2% glutaraldehyde for 10 minutes and stained with crystal violet for 1 hour at room temperature with shaking. Following staining, the plates were dried, and the dye was eluted with acetic acid. Absorbance was measured at 590 nm using an AGILENT Biotek Synergy H1 multiplate reader.

### Tumor growth and drug response *in vivo*

Female immunocompromised NMRI-Foxn1^nu/nu^ mice (Janvier Labs), typically 6 weeks old, were used to generate xenografts. Fresh patient-derived xenograft (PDX)-derived tumor fragments were implanted subcutaneously or orthotopically, respectively. Mice were then treated daily with 20 mg/kg dasatinib or vehicle (30% PEG and 5% Tween-80 in physiologic serum) intraperitoneally. The treatment groups were randomized, and the health of the mice was monitored regularly. Tumor size was measured daily using digital calipers once the growth was observed.

## Results

### Identification of basal markers through integrated transcriptomic- and protein-level analyses of the healthy mammary gland

**Figure 1.**
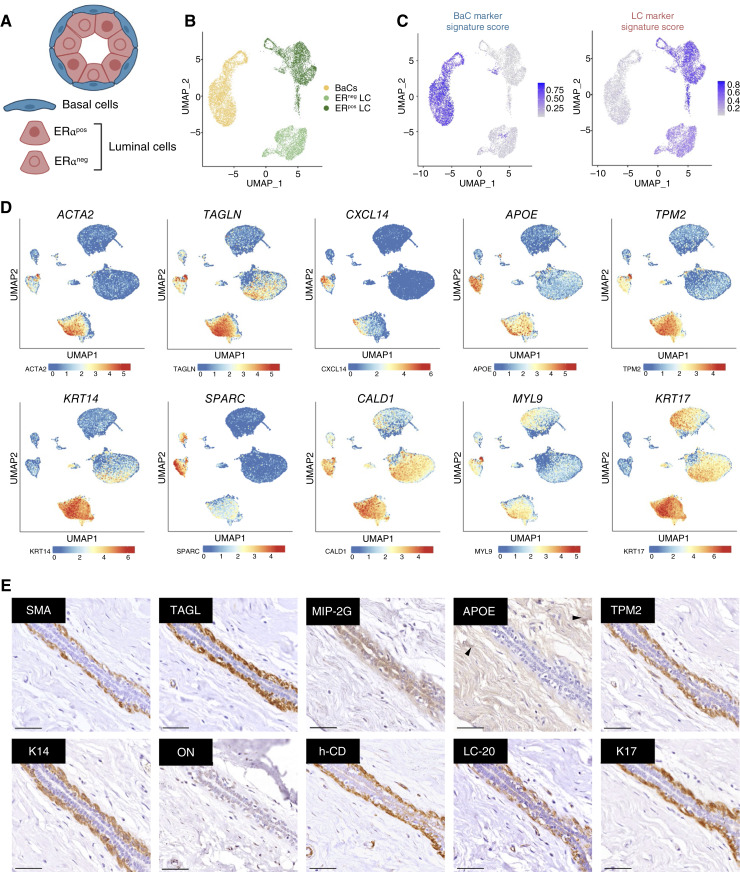
Identification of BaC identity markers expressed in the basal compartment of healthy human breast. **A,** Schematic representation of a mammary gland duct cross-section, showing BaCs (blue) and LCs cells (red). ERα^pos^ and ERα^neg^ statuses are indicated by nuclear color. **B,** Integrated UMAP plot showing epithelial cell clustering from three murine scRNA-seq datasets. Color coding differentiates between BaCs (blue), ERα^pos^ LC (red), and ERα^neg^ LCs (green). **C,** UMAP plots showing the signature score of the top 20 DEGs associated with each epithelial compartment. Signature scores are color-coded from low (gray) to high (blue). **D,** UMAP plots showing expression levels of the top 10 BaC-associated genes across the integrated human dataset. Color gradient indicates expression levels from low (blue) to high (red). **E,** Representative IHC staining of breast tissue consecutive serial sections showing proteins expression of candidate BaC-associated genes. Scale bar, 50 μm. Arrows in the APOE panel highlight expression restricted to stromal cells. **A,** Created in BioRender. Rodilla, V. (2026) https://BioRender.com/qpb7bux.

Publicly available scRNA-seq data from three studies of healthy murine mammary glands [GSE109711 (18), GSE164017 (19), and GSE148791 (20)] were integrated to generate unique epithelial signatures capable of distinguishing three epithelial clusters corresponding to basal and luminal (ERα^pos^ and ERα^neg^) cells (Fig. 1A and B; Supplementary Fig. S1A and S1B). Given that approximately 77% of TNBC tumors are classified as basal-like by PAM50 at the transcriptomic level (7), we first identified DEGs between BaCs and LCs to define candidate basal identity markers. To refine this selection, we ranked genes based on their loading along PC1, which clearly separates basal from luminal compartments (Supplementary Fig. S1C). The top 20 basal- and luminal-associated DEGs were selected (Fig. 1C; Supplementary Figs. S2 and S3).

Next, candidate basal markers were further refined based on their ability to consistently identify human BaCs at both the transcriptomic and protein levels. To confirm cross-species expression, we integrated three scRNA-seq datasets from healthy human breast tissues [GSE161529 (15), GSE180878 (21), and GSE113197 (14); Supplementary Fig. S4]. These analyses confirmed that the top 10 basal markers identified in murine tissues were also consistently expressed in human BaCs, supporting their value as conserved basal identity markers across species (Fig. 1D; Supplementary Fig. S2).

Although some markers, such as apolipoprotein E (Apo-E/*APOE*), C-X-C motif chemokine 14 (MIP-2G/*CXCL14*), and osteonectin (ON/*SPARC*), showed broader histologic expression beyond the basal compartment (Fig. 1E), protein-level validation confirmed that SMA/*ACTA2*, TAGL/*TAGLN*, TPM2, keratin 14 (K14), caldesmon (h-CD/*CALD1*), myosin regulatory light polypeptide 9 (LC-20/*MYL9*), and keratin 17 (K17) were exclusively expressed in BaCs located in the outer layer of the mammary epithelium in healthy human breast tissue (Fig. 1E). These results highlight a robust set of basal markers with conserved transcriptomic- and protein-level expression that define the basal epithelial identity in the healthy mammary gland.

### Selection of robust basal markers in TNBC

**Figure 2.**
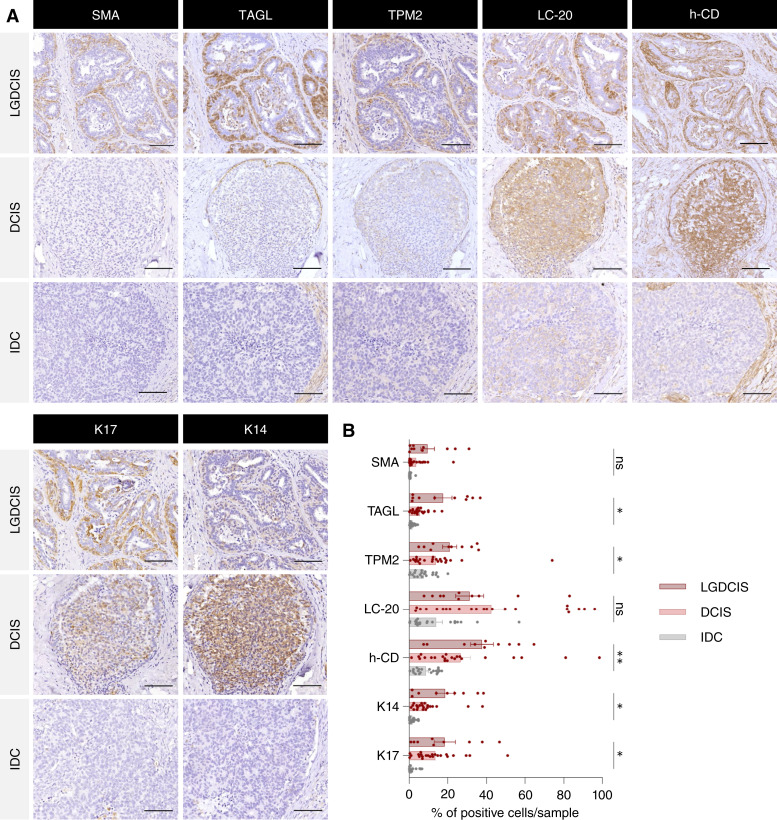
Histologic expression of basal markers in HR-positive breast tumors at different tumor stages. **A,** Representative sections of HR-positive samples at different tumor stages (LGDCIS, DCIS, and IDC) stained with the indicated basal markers. Scale bar, 50 μm. **B,** Bar plots representing the percentage of cells expressing each marker in in HR-positive samples across the different tumor stages. Statistics calculated two-way ANOVA. *, *P* < 0.05; **, *P* < 0.005; ns, not significant.

**Figure 3.**
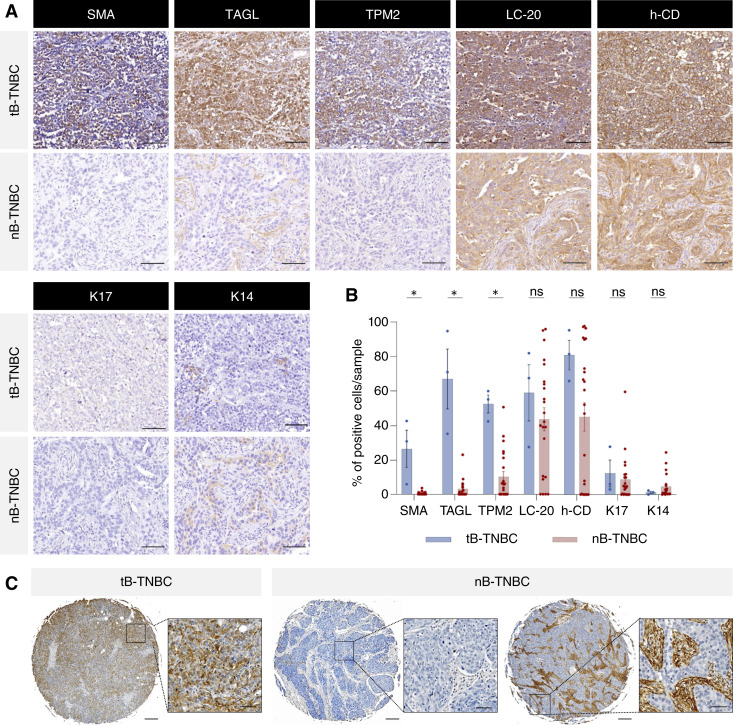
Histologic expression of basal markers in breast tumor samples. **A,** Representative sections of tB- and nB-TNBC samples stained with the indicated BaC markers. Scale bar, 50 μm. **B,** Bar plots representing the percentage of cells expressing each marker in tB- and nB-TNBCs. *, *P* < 0.05 based on unpaired *t* test (Holm–Sidak method); ns, not significant. **C, **Left, representative images showing TAGL staining in tB- and nB-TNBC tumors. Scale bar, 200 and 50 μm in the insets.

The specificity of these seven basal markers identified in the healthy mammary gland was evaluated in a discovery cohort comprising 54 HR-positive tumors at different stages, including LGDCIS, DCIS, and IDC, and 26 TNBC samples collected at diagnosis. In LGDCIS, basal marker expression was restricted to the myoepithelial layer surrounding the tumor (Fig. 2A). In contrast, expression was nearly absent in advanced IDC samples (Fig. 2B), indicating progressive loss of the basal layer during HR-positive disease evolution. In DCIS, most markers remained confined to nontransformed myoepithelial cells, except for LC-20 and h-CD, which were also detected in tumor cells in more than 25% of cases (Fig. 2B), suggesting that certain basal markers may be reactivated in LCs during tumor progression. We also evaluated the expression of these seven basal markers in the TNBC samples. Unsupervised hierarchical clustering based on the percentage of positive tumor cells for each marker stratified the tumors into two distinct groups: tB-TNBC and nB-TNBC (Supplementary Fig. S5). SMA, TAGL, TPM2, LC-20, and h-CD were all expressed in the tB-TNBC group (Fig. 3A; Supplementary Fig. S5), which represented approximately 11.5% of cases (3/26). Notably, K14 and K17 were poorly expressed (<10% of positive cells) in TNBC samples (Fig. 3B; Supplementary Fig. S5), suggesting that expression of these cytokeratins is lost during tumorigenesis. Based on these observations, LC-20 and h-CD were excluded because of their lack of specificity, and K14 and K17 were discarded because of low expression in TNBC. Through this bottleneck strategy, SMA, TAGL, and TPM2 were selected as robust and specific markers of basal identity. Interestingly, in nB-TNBC tumors, basal marker expression was restricted to stromal cells, with no staining detected in tumor epithelial cells (Fig. 3C; Supplementary Fig. S5).

**Figure 4.**
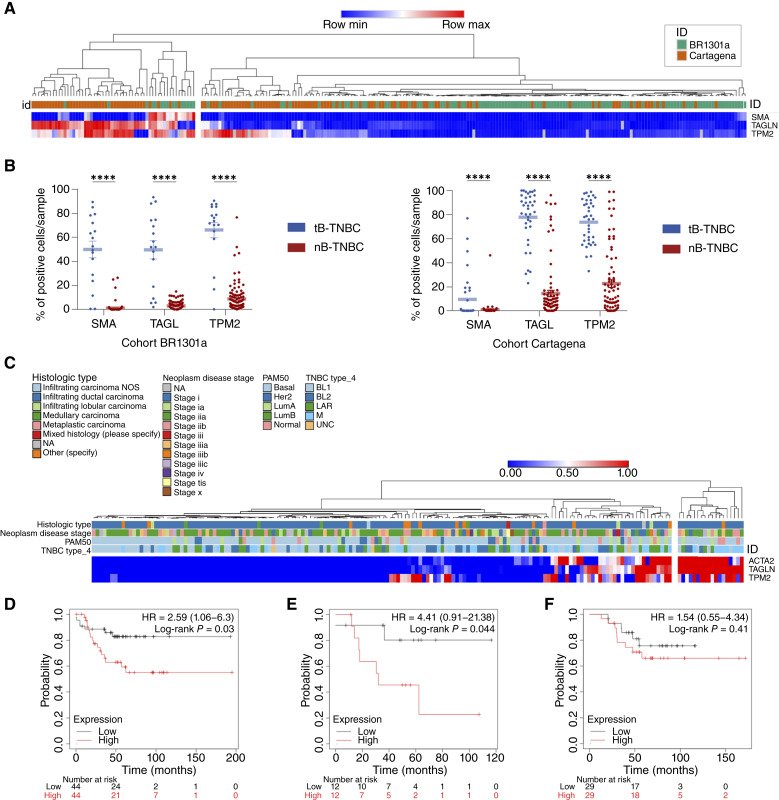
Expression and prognostic impact of tB-markers in TNBC. **A,** Unsupervised hierarchical clustering of the percentage of positive cells/sample in the discovery and validation cohorts of TNBC (*n* = 243). Color code denotes percentage of expression (blue, 0%; red, 100%). **B,** Scatter plots representing the percentage of tumor cells positive for SMA, TAGL, and TPM2 in two independent TNBC cohorts: the commercial TMA BR1301a (left) and the Cartagena cohort (right). Each dot represents one patient sample; horizontal lines indicate mean ± SEM. Statistical significance was determined using two-way ANOVA. ****, *P* < 0.0001. **C,** Heatmap showing the expression levels of the tB-markers across breast cancer samples. Expression values are row-normalized, ranging from low (blue) to high (red). The top annotations indicate relevant clinical and molecular characteristics of each sample, including histologic type, disease stage, intrinsic PAM50 subtype, and Lehmann classification (TNBC type_4). NA, not available; NOS, not otherwise specified. **D,** KM plot depicting RFS of patients with TNBC stratified by tB-marker expression. **E,** KM plot showing RFS in patients with TNBC treated exclusively with chemotherapy, based on tB-marker expression. **F,** KM plot illustrates OS of patients with TNBC based on tB-marker expression. In panels **D–F**, patient subgroups were defined through trichotomization, in which the lower quartile represents the “low expression” group (black), and the upper quartile represents the “high expression” group (red). Patients with intermediate expression levels were excluded to ensure a robust comparison between high and low expressers.

To validate their diagnostic value, we performed IHC on two independent TNBC validation cohorts: a commercial TMA (BR1301a, *n* = 120) and a cohort from Hospital General Universitario Santa Lucía (Cartagena, Spain; *n* = 123). Unsupervised hierarchical clustering of marker expression again stratified the samples into two distinct groups (tB-TNBC and nB-TNBC; Fig. 4A). In parallel, we established marker-specific thresholds using a standard biomarker evaluation approach. Histogram plots of marker expression revealed bimodal distributions, from which the valley between peaks was used as the positivity cutoff, corresponding to >5% for SMA, >30% for TAGL, and >40% for TPM2 (Supplementary Fig. S6A). Notably, the classification based on these thresholds matched the unsupervised clustering results, reinforcing the biological consistency of the subclassification. Furthermore, tumors classified as tB-TNBC consistently expressed at least two of the three tB-markers above their respective cutoffs, even though no such combinatorial rule was preimposed (Fig. 4B). This convergence across two independent classification strategies further supports the robustness of our approach. Based on these criteria, 22.6% of TNBC cases (55/243) in the validation cohorts were classified as tB-TNBC (Fig. 4A; Supplementary Tables S1 and S2). The diagnostic value of the three tB-markers was further assessed using receiver operating characteristic curve analysis, which showed excellent discriminatory ability for TAGL (AUC = 0.91 ± 0.01) and TPM2 (AUC = 0.95 ± 0.01) and moderate performance for SMA (AUC = 0.73 ± 0.02; Supplementary Fig. S6B).

To explore whether this marker-based classification is also reflected at the transcriptomic level, we analyzed TNBC samples from TCGA (28). Approximately 10.2% of tumors (18/176) were classified as tB-TNBC based on high expression of *ACTA2*, *TAGLN*, and *TPM2*. Importantly, these markers did not cluster with any particular PAM50 or Lehmann subtypes (Fig. 4C), supporting the notion that tB-TNBC represents a biologically distinct subgroup not captured by existing classification schemes.

To further characterize the molecular identity of this subgroup, we performed differential expression analysis between tB-TNBC and nB-TNBC tumors in the TCGA cohort (28) using expression of *ACTA2*, *TAGLN*, and *TPM2* as classifiers. The resulting tB-TNBC gene signature included these three markers as the most significantly upregulated genes, *TAGLN* ranking first, further validating their subclassification value (Supplementary Fig. S6C). Finally, to explore whether this transcriptional program mirrors that of normal basal epithelial cells, we compared the tB-TNBC signature with the basal identity genes previously identified from scRNA-seq of healthy mammary glands. This comparison revealed a shared set of 23 genes (Supplementary Fig. S6D), including cytoskeletal components, extracellular matrix regulators, and contractility-associated transcripts. These findings suggest the existence of a conserved basal program that is active in both normal and malignant BaCs.

Taken together, these results confirm that *ACTA2*, *TAGLN*, and *TPM2* are reliable and specific biomarkers for identifying a distinct TNBC subgroup, termed as tB-TNBC, at both the transcriptomic and protein levels. Their consistent expression defines a previously unrecognized TNBC subtype, providing new insights into tumor heterogeneity and enabling more precise patient stratification.

### Clinical characterization and prognostic value of tB-markers in TNBC

To determine whether tB- and nB-TNBC differ in their clinical features, we analyzed key pathologic parameters across both validation cohorts. Overall, no statistically significant differences were observed in tumor size, nodal involvement, metastatic status, histologic subtype, or tumor grade. Although some variability was noted, such as a slightly older mean age at diagnosis in the tB-TNBC group, these differences did not reach statistical significance and were not consistently replicated across cohorts. These findings indicate that tB-TNBC cannot be reliably identified based on conventional clinicopathologic criteria (Supplementary Tables S1 and S2), underscoring the relevance of molecular biomarkers for accurate TNBC subclassification.

Additionally, to evaluate the clinical impact of the tB-markers, we used the KM plotter tool to correlate their expression with patient outcomes across breast cancer datasets. Stratifying patients by the mean expression of *ACTA2*, *TAGLN*, and *TPM2* (upper vs. lower quartiles), we found that high expression was associated with a significantly worse prognosis, RFS with a HR of 2.59 (Fig. 4D). Moreover, in patients treated exclusively with chemotherapy, prognosis was even poorer (HR = 4.41, Fig. 4E), suggesting that tB-TNBC tumors may derive limited benefit from standard chemotherapy regimens. No significant differences were observed in OS (HR = 1.54, Fig. 4F). These findings were corroborated using external datasets via bc-GenExMiner, in which high expression of each tB-marker individually was associated with reduced disease-free and metastasis-free survival (Supplementary Fig. S7).

To further explore the therapeutic implications of the tB-TNBC subtype, we examined the relationship between tB-marker expression and response to immune checkpoint inhibitors. Using transcriptomic data from the phase II I-SPY2 clinical trial (GSE173839; ref. 38), in which patients with TNBC received neoadjuvant durvalumab (anti–PD-L1), olaparib, and paclitaxel, we observed significantly higher expression of *ACTA2*, *TAGLN*, and *TPM2* in nonresponders (non-pCR) compared with responders (pCR; Supplementary Fig. S8A). These findings suggest that tB-TNBC may be less likely to benefit from anti–PD-L1–based immunotherapy, as well as from standard chemotherapy regimens (Fig. 4E; Supplementary Fig. S8A). To determine whether this poor response reflects an immunologically inert tumor microenvironment, we analyzed the expression of key immune checkpoint–related genes (*PDCD1*, *CD274*, *CTLA4*, *TIGIT*, and *PVR*) in the TCGA cohort (*n* = 176; ref. 28), stratified by tB-TNBC status. tB-TNBC tumors exhibited consistently low or absent expression of these immune markers, indicating an immunologically “cold” phenotype (Supplementary Fig. S8B). Collectively, these results suggest that tB-TNBC tumors may not respond to immune checkpoint blockade targeting the PD-1/PD-L1 or CTLA4/CD80 axes, highlight the need for alternative therapeutic strategies tailored to this biologically distinct and immunoresistant subtype.

In summary, our results demonstrate that *ACTA2*, *TAGLN*, and *TPM2* are not only robust markers of basal identity but also serve as reliable biomarkers for identifying a clinically relevant TNBC subgroup. This molecular subclassification captures biologically meaningful heterogeneity within TNBC and may provide valuable insights for prognosis and therapeutic decision-making.

### Identification of therapeutic compounds for the targeted treatment of tB-TNBC tumors

**Figure 5.**
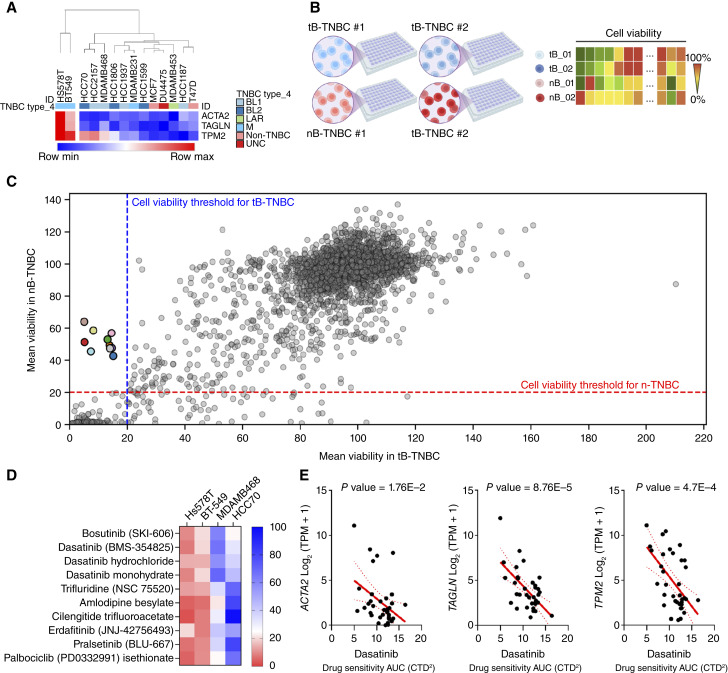
Identification of candidate compounds selectively targeting tB-TNBC cells. **A,** Heatmap showing the transcriptomic expression of tB-markers in a panel of breast cancer cell lines. **B,** Schematic representation of the FDA-approved drug library screening workflow. Cell viability heatmap shows the response of each cell line to the 3,200 tested compounds. ***, *P* < 0.001. **C,** Dot plot of average cell viability (%) in tB-TNBC (*x*-axis) and nB-TNBC (*y*-axis) cell lines after treatment. Dotted red and blue lines indicate the viability thresholds used for compound selection (<20% viability in tB-TNBC and >20% in nB-TNBC). Colored dots highlight the selective compounds. **D,** Heatmap showing the viability percentages of the selected compounds across the four TNBC cell lines used in the screen. **E,** Correlation plots showing the association between *ACTA2*, *TAGLN*, and *TPM2* expression (log_2_ TPM + 1) and dasatinib sensitivity (AUC) in breast cancer cell lines. Red lines, regression fit; shaded areas show 95% confidence intervals. *P* values calculated by Pearson correlation. **B,** Created in BioRender. Rodilla, V. (2026) https://BioRender.com/qpb7bux.

To identify therapeutic strategies specifically effective against tB-TNBC, we performed high-throughput screening of 3,200 FDA-approved compounds using breast cancer cell lines stratified by expression of the tB-markers (*ACTA2*, *TAGLN*, and *TPM2*). From a panel of breast cancer lines, we selected two tB-TNBC models (Hs578T and BT-549) and two nB-TNBC models (HCC70 and MDA-MB-468) that exhibited low or undetectable tB-marker expression (Fig. 5A). Cells were seeded under standardized conditions to ensure reproducibility and treated with each compound at a fixed concentration of 10 μmol/L for 72 hours. Cell viability was then assessed using crystal violet staining (Fig. 5B and C). Compounds that reduced viability by >80% in both tB-TNBC cell lines while maintaining >20% viability in the nB-TNBC models were considered selectively effective and shortlisted for further analysis (Fig. 5C and D).

To prioritize compounds whose efficacy was specifically linked to tB-marker expression, we performed correlation analyses using public expression and drug sensitivity data from the *DepMap* portal. Specifically, we evaluated whether the cytotoxicity of each compound correlated with transcriptomic expression of *ACTA2*, *TAGLN*, and *TPM2* (Fig. 5E; Supplementary Fig. S9). Notably, dasatinib, a multi-kinase inhibitor approved for hematologic malignancies, showed a statistically significant correlation between drug sensitivity and tB-marker expression (Fig. 5E), suggesting potential selective efficacy in tB-TNBC models.

**Figure 6.**
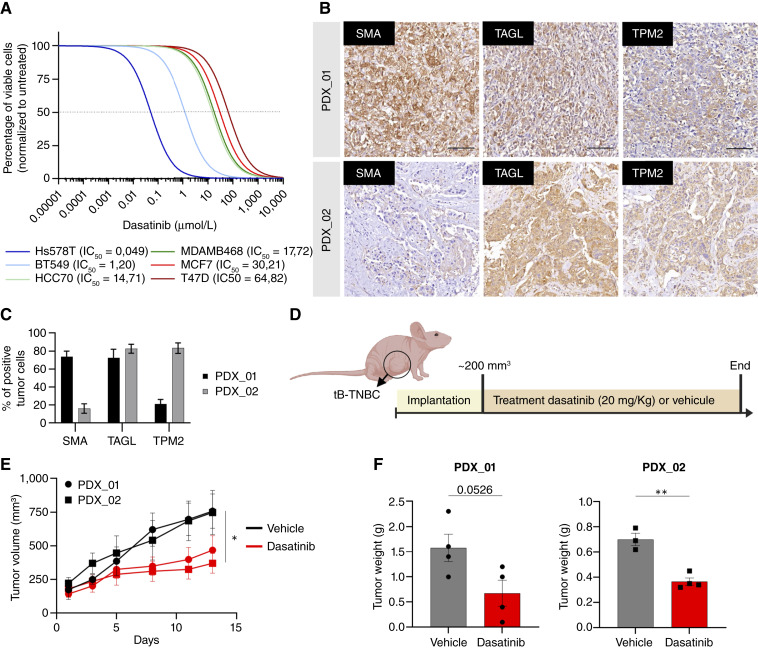
Evaluation of dasatinib sensitivity in tB-TNBC models *in vitro* and *in vivo*. **A,** Dose–response curves showing cell viability of indicated cell lines treated with increasing concentrations of dasatinib for 72 hours. Solid lines represent the mean of three biological replicates performed in technical replicates normalized to untreated controls, and IC_50_ values are indicated. **B,** Representative IHC images of tB-TNBC PDXs (PDX_01 and PDX_02) showing expression of SMA, TAGL, and TPM2. Scale bars, 100 μm. **C,** Quantification of positive tumor cells for each tB-marker in PDX_01 and PDX_02. Data are shown as mean ± SEM of different tumor areas. **D,** Schematic representation of the dasatinib treatment protocol. **E,** Tumor growth curves of the tB-TNBC PDXs treated with vehicle (black) or dasatinib (red). Error bars, ± SEM. *P* values calculated by Wilcoxon test. **F,** Bar plot showing tumor weight at the end point. Data are shown as mean ± SEM. *P* values calculated by two-tailed Student *t* test. **, *P* < 0.01. **D,** Created in BioRender. Rodilla, V. (2026) https://BioRender.com/qpb7bux.

To experimentally validate this finding, we performed dose–response assays in the same panel of TNBC cell lines. The tB-TNBC lines Hs578T and BT-549, which express high levels of tB-markers, showed markedly increased sensitivity to dasatinib (IC_50_ = 0.049 and 1.242 μmol/L, respectively), compared with the nB-TNBC lines HCC70 and MDA-MB-468 (IC_50_ = 15.40 and 18.23 μmol/L; Fig. 6A). As a negative control, HR-positive cell lines (MCF7 and T-47D) showed minimal sensitivity to dasatinib, consistent with their low tB-marker expression. To assess *in vivo* efficacy, we established PDX models positive for all three tB-markers (Fig. 6B and C). Mice bearing these PDX tumors were treated daily with dasatinib or vehicle for 2 weeks, and tumor growth was measured every other day (Fig. 6D and E). Dasatinib treatment significantly reduced tumor growth and final tumor weight compared with controls (Fig. 6E and F), demonstrating strong *in vivo* sensitivity of tB-TNBC tumors to this compound. Collectively, these results provide robust preclinical evidence supporting the repurposing of dasatinib as a subtype-specific therapy for tB-TNBC.

Furthermore, our findings highlight the utility of tB-markers not only for TNBC subclassification but also as predictive biomarkers of therapeutic response. These markers could serve as a stratification tool to identify patients most likely to benefit from dasatinib and potentially other targeted agents.

### The role of *TAGLN* in mediating dasatinib responsiveness in tB-TNBC

**Figure 7.**
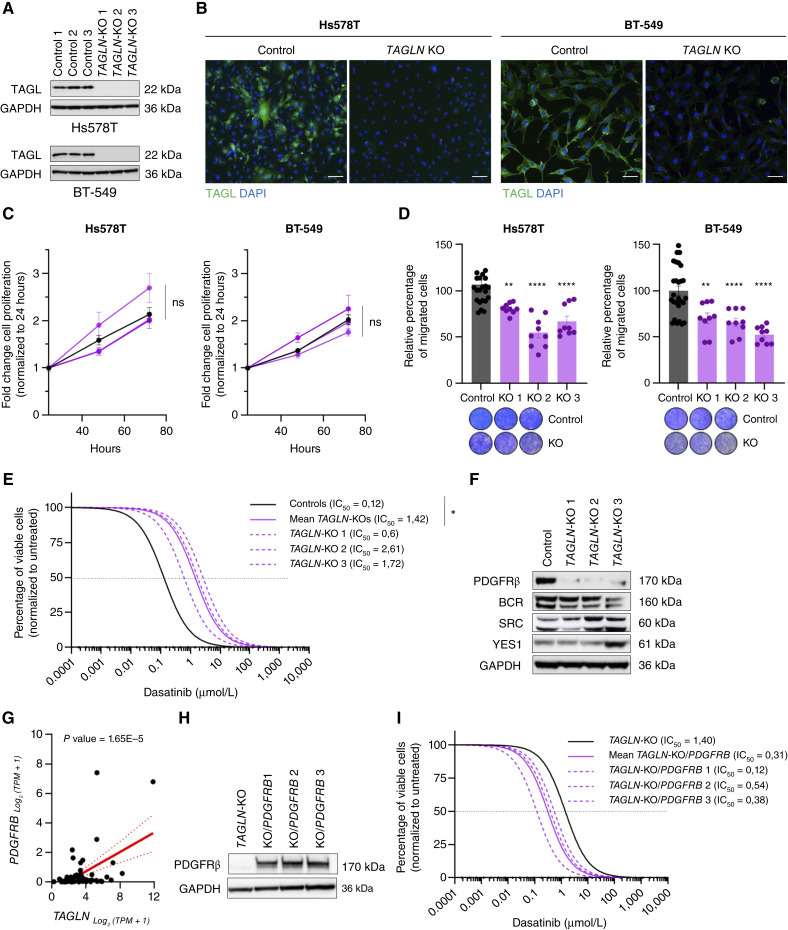
TAGL as a predictive marker for dasatinib sensitivity. **A,** Western blot of control and *TAGLN*-KO cells in Hs578T (top) and BT-549 (bottom) cells. **B,** Representative immunofluorescence of Hs578T and BT549 control and *TAGLN*-KO cells, stained with αTAGL (green) and DAPI (blue). Scale bar, 50 μm. **C,** Cell proliferation in control (black) and *TAGLN*-KO (purple) cells measured by MTT assays over 72 hours. **D,** Relative percentage of migrated cells in control and *TAGLN*-KO cells, assessed by transwell assays. In **C** and **D**, values represent the mean ± SEM of at least three experimental replicates, and statistical significance was determined using one-way ANOVA. **E,** Drug–response curves for cell viability of control and *TAGLN*-KO cells treated with dasatinib at increasing concentrations. **F,** Western blot of known dasatinib targets in control and *TAGLN*-KO cells. **G,** Data from the *DepMap* portal showing the correlation between *TAGLN* and *PDGFRB* in breast cancer cell lines. **H,** Western blot of PDGFRβ in *TAGLN*-KO and cells and clones constitutively expressing *PDGFRB*. **I,** Drug–response curves for cell viability of *TAGLN*-KO and *TAGLN*-KO/*PBGFRB* cells treated with dasatinib at increasing concentrations. In **E** and **I**, solid lines represent the mean of three biological replicates performed in technical replicates. The dashed line indicates their IC_50_ value. **, *P* < 0.01; ****, *P* < 0.0001; ns, not significant.

To determine whether tB-marker expression directly modulates sensitivity to dasatinib, we focused on *TAGLN*, which exhibited the most consistent and specific expression across tB-TNBC samples (Figs. 2A, 4A, and 6C). Based on this observation, we generated *TAGLN*-KO clones in two representative tB-TNBC cell lines, Hs578T and BT-549 (Fig. 7A and B), both selected for their high expression of tB-markers within a broader panel of breast cancer cell lines (Supplementary Fig. S10A).

Functional assays revealed that *TAGLN* is not essential for cell proliferation, as its depletion did not significantly affect the growth of either cell line (Fig. 7C). However, given *TAGLN*’s role as an actin-binding protein involved in cytoskeletal organization (39, 40), we next examined its impact on cell migration. *TAGLN*-KO cells displayed markedly reduced migratory capacity in both Hs578T and BT-549 compared with control cells (Fig. 7D), suggesting that *TAGLN* contributes to the migratory, and potentially metastatic, behavior of tB-TNBC cells.

To further investigate *TAGLN*-dependent pathways, we performed bulk RNA-seq on *TAGLN*-KO and control clones (Supplementary Fig. S10B). Surprisingly, few DEGs were shared between the two KO models (Supplementary Fig. S10C), indicating cell line–specific transcriptional responses. Nevertheless, GSEA revealed consistent disruption of key oncogenic pathways, including β-catenin and KRAS signaling (Supplementary Fig. S10D). Notably, the epithelial-to-mesenchymal transition (EMT) signature was significantly downregulated in BT-549 *TAGLN-*KO cells (Supplementary Fig. S10E), supporting a phenotypic shift toward a more epithelial-like, less migratory state (Fig. 7D).

We next examined whether *TAGLN* modulates dasatinib response. Dose–response assays showed that *TAGLN*-KO cells were significantly less sensitive to dasatinib, with increased IC_50_ values in both Hs578T (1.42 vs. 0.12 μmol/L) and BT-549 (4.315 vs. 0.74 μmol/L) cells (Fig. 7E; Supplementary Fig. S10F). To explore the underlying mechanism, we analyzed the expression of known dasatinib targets, including PDGFRβ, BCR, SRC, and YES (Supplementary Fig. S11A and S11B). Strikingly, only PDGFRβ was consistently downregulated upon *TAGLN* depletion (Fig. 7F), suggesting a functional link between TAGL and PDGFRβ regulation. Supporting this connection, publicly available datasets revealed a positive correlation between *TAGLN* and *PDGFRB* transcript levels across breast cancer cell lines (Fig. 7G). To functionally validate this relationship, we reexpressed *PDGFRB* in *TAGLN*-KO cells (Fig. 7H) and observed a partial restoration of dasatinib sensitivity (Fig. 7I).

Together, these results demonstrate that *TAGLN* modulates dasatinib responsiveness in tB-TNBC by regulating PDGFRβ expression, identifying TAGL as a key mediator of this subtype-specific therapeutic vulnerability.

## Discussion

TNBC is a biologically heterogeneous and clinically aggressive disease with limited targeted therapy options. In this study, we addressed this unmet clinical need by identifying and validating a novel set of basal-associated markers (SMA, TAGL, and TPM2) that define a distinct molecular subtype of TNBC, which we term tB-TNBC. Through integrated single-cell transcriptomics, IHC, and functional analyses, we demonstrate that this biomarker-defined subgroup exhibits specific biological and therapeutic vulnerabilities that are not captured by current transcriptomic classifiers such as PAM50 or Lehmann. It is important to note that basal-like tumors (as defined by PAM50 and Lehmann) typically exhibit enriched expression of classic basal cytokeratins such as *KRT5*, *KRT14*, and *KRT17*, which we showed are not specific to tB-TNBC tumors. Instead, our data suggests that many basal-like breast tumors may represent luminal TNBCs that acquire basal cytokeratin expression during tumor progression, highlighting the limitations of current classifiers in capturing functionally and therapeutically relevant TNBC heterogeneity. Importantly, we did not perform a direct comparison with the claudin-low subtype, which is not encompassed by these widely used classification systems. Claudin-low tumors have been previously associated with EMT-like features, immune infiltration, and low expression of tight junction proteins and share several traits with tB-TNBC, including mesenchymal and stem-like properties, making it plausible that the two subtypes partially overlap. However, due to the lack of unified and consistent claudin-low annotation across datasets, this comparison remains to be systematically explored. Future studies incorporating claudin-low–specific classifiers or molecular signatures will be necessary to determine whether tB-TNBC constitutes a novel subgroup or represents a refined stratification within this previously described phenotype.

Our study also underscores the importance of integrating single-cell transcriptomics with protein-level validation to refine biomarker selection. Using scRNA-seq data from healthy breast tissue, we initially identified a broad panel of basal-associated genes. To enrich for the most specific candidates, we prioritized genes by their contribution to PC1, which clearly separates BaCs from LCs, and selected those expressed in more than 75% of BaCs while excluding those with high expression in LCs (PC2 >50%). This filtering strategy drastically reduced the list to strong candidates (Supplementary Fig. S2). However, protein expression analysis revealed that some candidates, such as APOE, MIP-2G, and ON, lacked exclusive expression in BaCs in normal tissue samples. Interestingly, the expression of other basal markers, such as LC-20 and h-CD, in HR-positive tumors may reflect a reactivation of developmental programs or cellular plasticity. For instance, K14 expression has been reported in both luminal and basal compartments in aged breast tissue (21, 41), and luminal tumors with low ERα levels can acquire basal cytokeratin expression during progression (42). Moreover, collective invasion in luminal breast tumors seems to rely on K14-expressing leader cells (43). These findings suggest that whereas some basal markers may be reexpressed during tumor progression, others retain specificity and biological relevance as markers of tB identity.

Importantly, our results show that tB-TNBC cannot be identified through standard clinicopathologic parameters alone. Although no significant differences were observed between tB- and nB-TNBC tumors in terms of clinical or pathologic features across two independent patient cohorts, tB-markers consistently identified a distinct subgroup within the TNBC population. Moreover, whereas RFS was significantly shorter in patients with high tB-marker expression, OS was not, likely due to the influence of subsequent lines of therapy. Further validation using an independent dataset from the bc-GenExMiner platform confirmed that high *ACTA2*, *TAGLN*, and *TPM2* expression was significantly associated with reduced disease-free and distant metastasis-free survival. The lack of association with OS may reflect the multifactorial nature of this endpoint, which is affected by treatment response heterogeneity and non–cancer-related mortality. In contrast, recurrence-based endpoints are more directly influenced by the intrinsic tumor features captured by the tB-markers. Together, these results support the prognostic value of these markers and reinforce their robustness across datasets.

We further show that tB-markers are not merely phenotypic indicators but contribute to tumor behavior. Among them, *TAGLN*, the most consistently expressed, plays a functional role in supporting cell migration, potentially through regulation of EMT and PDGFRβ signaling. *TAGLN* loss impaired migration *in vitro* and was associated with a shift toward an epithelial-like transcriptomic state, reinforcing its role in maintaining mesenchymal traits. However, when comparing transcriptomic profiles across *TAGLN*-KO lines in different tB-TNBC models, few shared DEGs were found, suggesting context-specific transcriptional consequences. Interestingly, the role of *TAGLN* in tumor progression has also been explored in other tumor types, including its involvement in cell proliferation, migration, and inhibition of tumor formation in colorectal cancer (39), non–small cell lung cancer (44) and lung adenocarcinoma (45).

To translate these findings into therapeutic insights, we performed drug screening using an FDA-approved compound library. This approach identified dasatinib as selectively cytotoxic to tB-TNBC cell lines while sparing nB-TNBC models. The association between tB-marker expression and dasatinib sensitivity was confirmed both *in vitro* and *in vivo* and through drug–gene correlation data from *DepMap* potal. These findings position *TAGLN* not only as a marker of basal identity but also as a functional mediator of therapeutic response. Moreover, *TAGLN* KO conferred resistance to dasatinib through downregulation of PDGFRβ, and rescue of *PDGFRB* expression restored drug sensitivity, providing mechanistic evidence for this regulatory link. Crucially, previous studies in ovarian cancer support a connection between *TAGLN* and Src signaling, another dasatinib target, suggesting a broader regulatory role (46). Specifically, ovarian cancer cells treated with dasatinib exhibited a substantial reduction in TAGL expression and Src activation, and *TAGLN* silencing led to decreased Src activation. Conversely, ectopic expression of Src active forms or TAGL overexpression increases TAGL and Src activation, respectively (46). Altogether, our results suggest that TAGL may participate in a regulatory loop involving multiple dasatinib-associated targets, strengthening its potential as a predictive biomarker.

Our findings may help explain the limited clinical efficacy of dasatinib observed in unstratified TNBC trials (NCT00817531 and NCT00371254), in which ∼10% of patients showed benefit, closely matching the frequency of tB-TNBC observed in our cohorts. This reinforces the need for biomarker-driven patient stratification in clinical trials. Besides, analysis of publicly available trial data (I-SPY2) and transcriptomic datasets revealed that tB-TNBC tumors are associated with poor response to anti–PD-L1 immunotherapy, suggesting that tB-TNBC may constitute an immunologically “cold” subtype. Although our immune-related analyses are exploratory and limited by dataset availability, they support the urgent need to explore alternative therapeutic strategies, such as dasatinib, for this patient population and highlight the relevance of molecular subclassification for guiding immunotherapy decisions.

Taken together, our findings provide compelling evidence for the clinical relevance of SMA, TAGL, and TPM2 in TNBC. These tB-markers offer a new stratification strategy that goes beyond current transcriptomic classifiers, delivering both prognostic and predictive insights. Despite certain limitations, such as sample size in early-stage analyses, the context-dependency of transcriptomic responses, and the need for broader clinical validation, our integrative approach defines a clinically actionable TNBC subgroup with distinct vulnerabilities. By identifying a subset of TNBCs susceptible to dasatinib and potentially other targeted therapies, this work sets the groundwork for biomarker-guided precision oncology in a patient population historically underserved by current therapeutic options.

## Supplementary Material

Supplementary Figure S1 Integration of three scRNA-seq datasets from adult murine mammary gland

Supplementary Figure S2 Expression of BaC-associated genes in murine mammary epithelial subpopulations

Supplementary Figure S3 Expression of LC-associated genes in murine mammary epithelial subpopulations

Supplementary Figure S4 Integration of three scRNA-seq datasets from healthy human mammary gland

Supplementary Figure S5 Immunohistochemical validation of basal markers in TNBC samples

Supplementary Figure S6 Histological distribution of SMA, TAGL and TPM2 in the validation cohorts of TNBC

Supplementary Figure S7 Prognostic value of ACTA2, TAGLN, and TPM2

Supplementary Figure S8 Analysis of basal marker expression and immune checkpoint genes in TNBC

Supplementary Figure S9 Correlation between ACTA2, TAGLN, and TPM2 expression and drug sensitivity of selected compounds of drug screening

Supplementary Figure S10 Functional validation of TAGLN-KO cells

Supplementary Figure S11 Analysis of TAGLN expression and dasatinib target interaction in cancer cell lines

Supplementary Table S1 Distribution of clinical and pathological features in the validation cohort BR1301a

Supplementary Table S2 Distribution of clinical and pathological features in the validation cohort of Hospital Universitario Santa Lucía, Cartagena

Supplementary Table S3 Primary antibodies used for immunohistochemistry

Supplementary Table S4 Secondary antibodies used for IHC, IF, and WB assays

Supplementary Table S5 Primary antibodies used in IF and WB assays

## Data Availability

The datasets analyzed during the current study are available in the Gene Expression Omnibus (GEO) repository under the accession numbers GSE109711, GSE164017, GSE148791, GSE161529, GSE180878, GSE113197, and GSE173839. The data generated in this study are publicly available in GEO at GSE300385. Data from the TCGA BRCA cohort were retrieved from the FireBrowse portal (http://firebrowse.org/). The bulk RNA-seq analysis pipeline used in this study is publicly available on the Galaxy platform: the workflow for quality control, mapping, and counting can be accessed at https://usegalaxy.eu/u/dolivares_ijc/w/qc--mapping--counting---ref-based-rna-seq---transcriptomics---gtn---subworkflows, the workflow for DEA and GSEA is available at https://usegalaxy.eu/published/workflow?id=888d545038843b5f, and the scripts of the scRNA-seq analysis can be found at https://github.com/mereulab/TNBC_study/tree/main/human. All other raw data generated in this study are available upon request to the corresponding author.
