# Supplementary material for: Discovery and Evaluation of Biomarkers for Triple-Negative Breast Cancer Subtypes Uncovers Patient Stratification and Targeted Therapeutic Strategies
Source: Cancer Res. 2026 Feb 11;86(10):2360–76. doi: 10.1158/0008-5472.CAN-24-2758 (PMC13176827; doi:10.1158/0008-5472.CAN-24-2758)
Supplement: Supplementary Figure S1 — Integration of three scRNA-seq datasets from adult murine mammary gland [file can-24-2758_supplementary_figure_s1_suppsf1.pdf]

Supplementary Figure S1

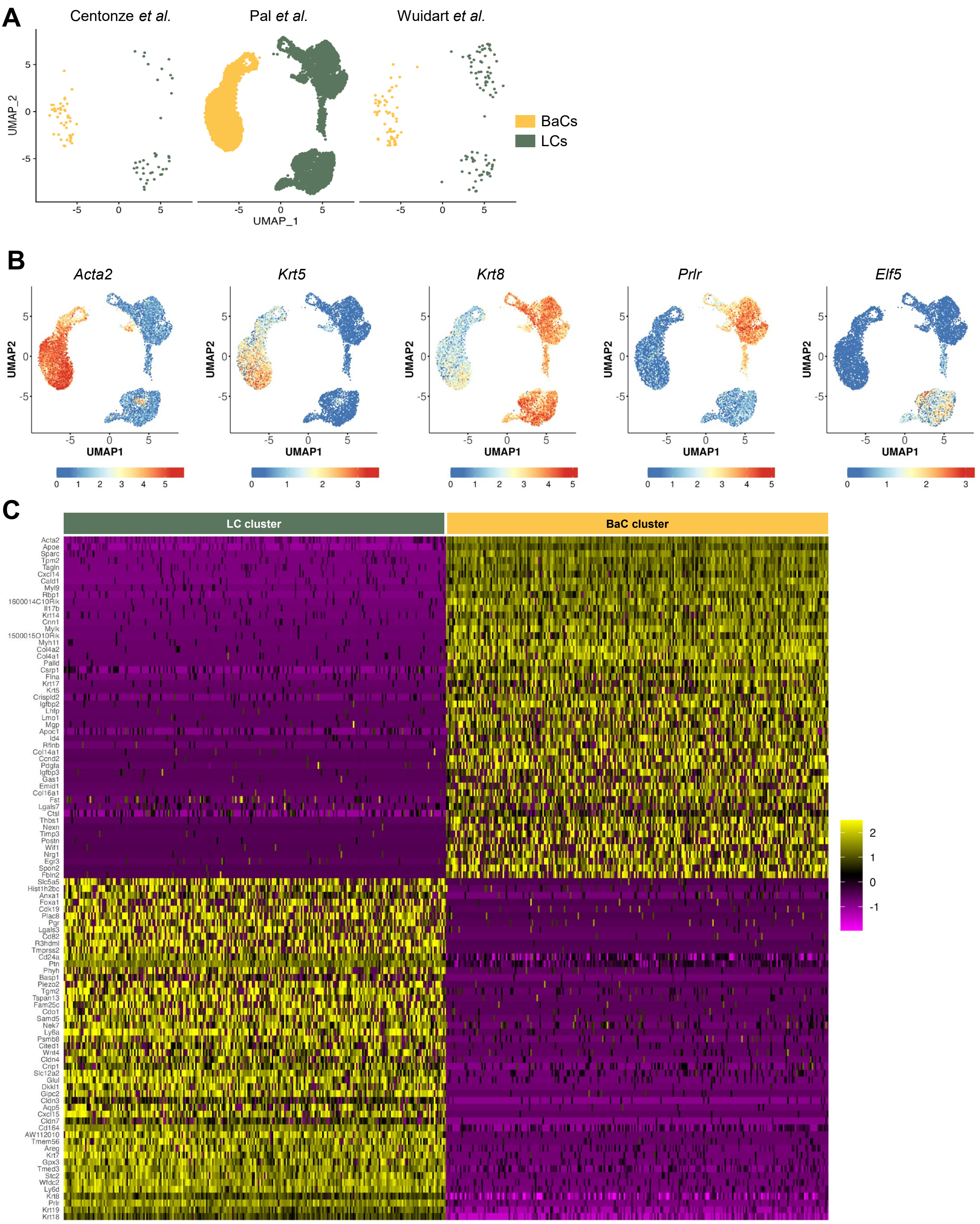

Supplementary Figure S1 | Integration of three scRNA-seq datasets from adult murine mammary gland. A, UMAP plots showing the distribution of epithelial cell populations from three independent studies [18-20]. Each dot represents a single cell, and colors distinguish between BaCs (dark green) and LCs (yellow). **B,** UMAP plots of canonical marker gene expression used to annotate epithelial clusters in the integrated dataset. *Krt5* and *Acta2* identify BaCs; *Elf5* and *Krt8* characterize ERα-neg LCs; and *Prlr* and *Krt8* define ERα-pos LCs. Color gradients indicate gene expression levels from low (blue) to high (red). **C**, Heatmap of the top 100 genes contributing to principal component 1 (PC1) variance across 500 cells. Each row corresponds to a gene, and each column to a single cell. Color intensity indicates normalized gene expression levels, ranging from -1 (purple) to +2 (yellow).
