# Supplementary material for: Discovery and Evaluation of Biomarkers for Triple-Negative Breast Cancer Subtypes Uncovers Patient Stratification and Targeted Therapeutic Strategies
Source: Cancer Res. 2026 Feb 11;86(10):2360–76. doi: 10.1158/0008-5472.CAN-24-2758 (PMC13176827; doi:10.1158/0008-5472.CAN-24-2758)
Supplement: Supplementary Figure S2 — Expression of BaC-associated genes in murine mammary epithelial subpopulations [file can-24-2758_supplementary_figure_s2_suppsf2.pdf]

Supplementary Figure S2

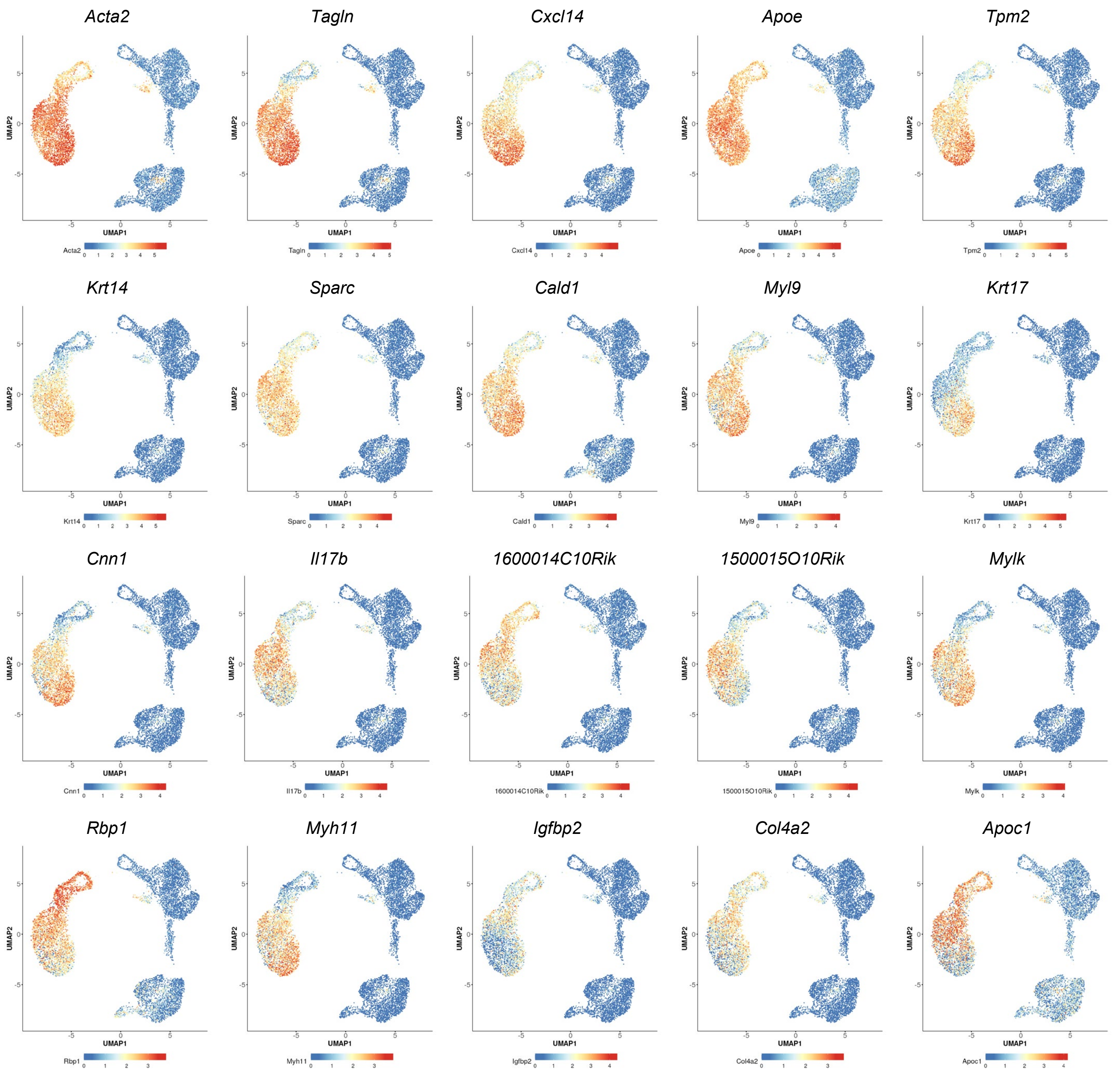

Supplementary Figure S2 | Expression of BaC-associated genes in murine mammary epithelial subpopulations. This figure presents a series of UMAP plots illustrating the expression patterns of the Top 20 BaC-associated markers across integrated scRNA-seq data sets. Each plot corresponds to a specific gene, as indicated at the top of each plot. Points within the plots represent individual cells, with the color gradient indicating gene expression levels from low (blue) to high (red).
