# Supplementary material for: Discovery and Evaluation of Biomarkers for Triple-Negative Breast Cancer Subtypes Uncovers Patient Stratification and Targeted Therapeutic Strategies
Source: Cancer Res. 2026 Feb 11;86(10):2360–76. doi: 10.1158/0008-5472.CAN-24-2758 (PMC13176827; doi:10.1158/0008-5472.CAN-24-2758)
Supplement: Supplementary Figure S3 — Expression of LC-associated genes in murine mammary epithelial subpopulations [file can-24-2758_supplementary_figure_s3_suppsf3.pdf]

Supplementary Figure S3

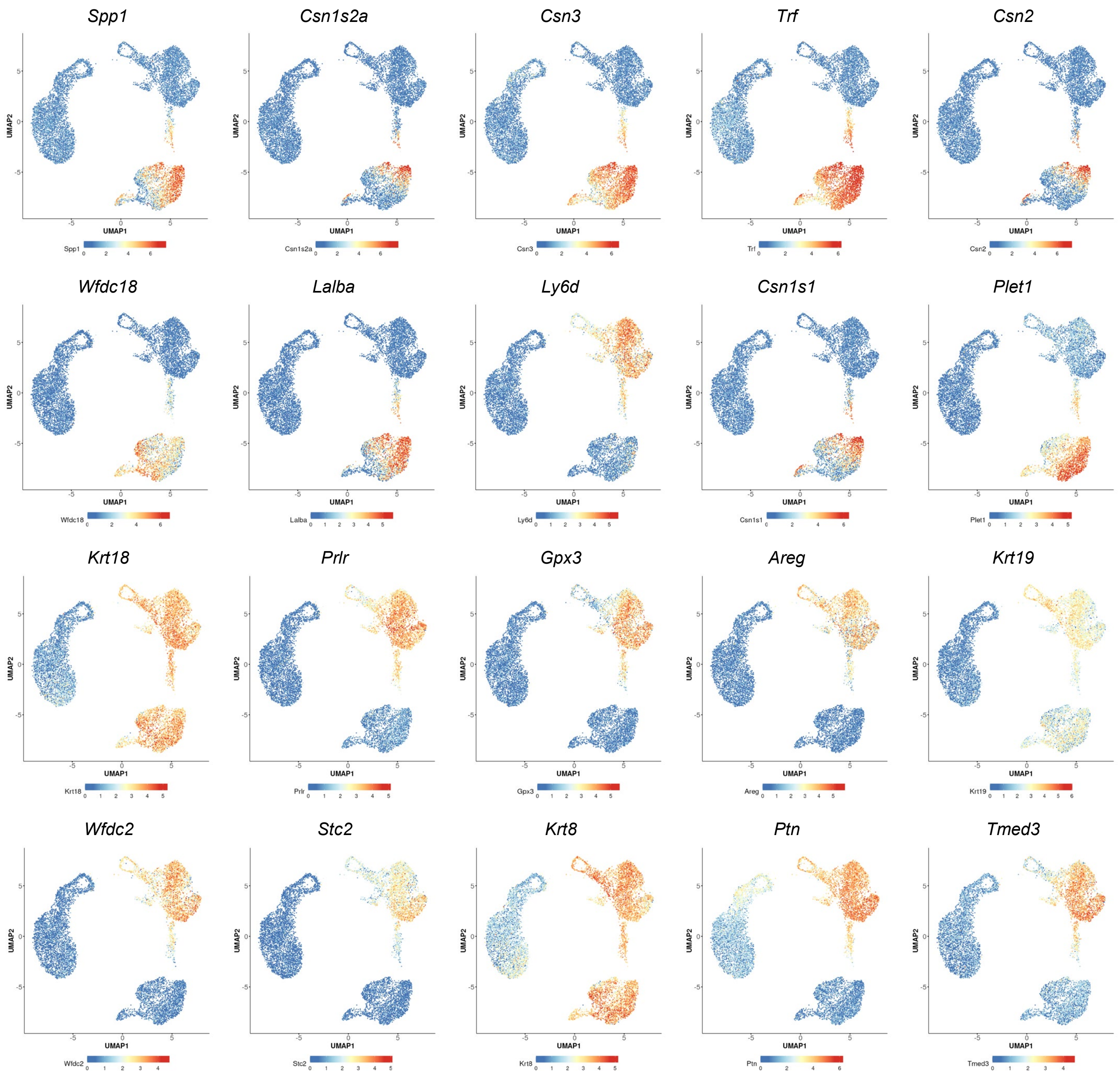

Supplementary Figure S3 | Expression of LC-associated genes in murine mammary epithelial subpopulations. This figure displays a series of UMAP plots depicting the expression patterns of the top 20 LC-associated markers across integrated scRNA-seq datasets. Each plot corresponds to a specific gene, as labeled at the top of each plot. Points within the plots represent individual cells, with the color gradient indicating gene expression levels ranging from low (blue) to high (red).
