# Supplementary material for: Discovery and Evaluation of Biomarkers for Triple-Negative Breast Cancer Subtypes Uncovers Patient Stratification and Targeted Therapeutic Strategies
Source: Cancer Res. 2026 Feb 11;86(10):2360–76. doi: 10.1158/0008-5472.CAN-24-2758 (PMC13176827; doi:10.1158/0008-5472.CAN-24-2758)
Supplement: Supplementary Figure S4 — Integration of three scRNA-seq datasets from healthy human mammary gland [file can-24-2758_supplementary_figure_s4_suppsf4.pdf]

Supplementary Figure S4

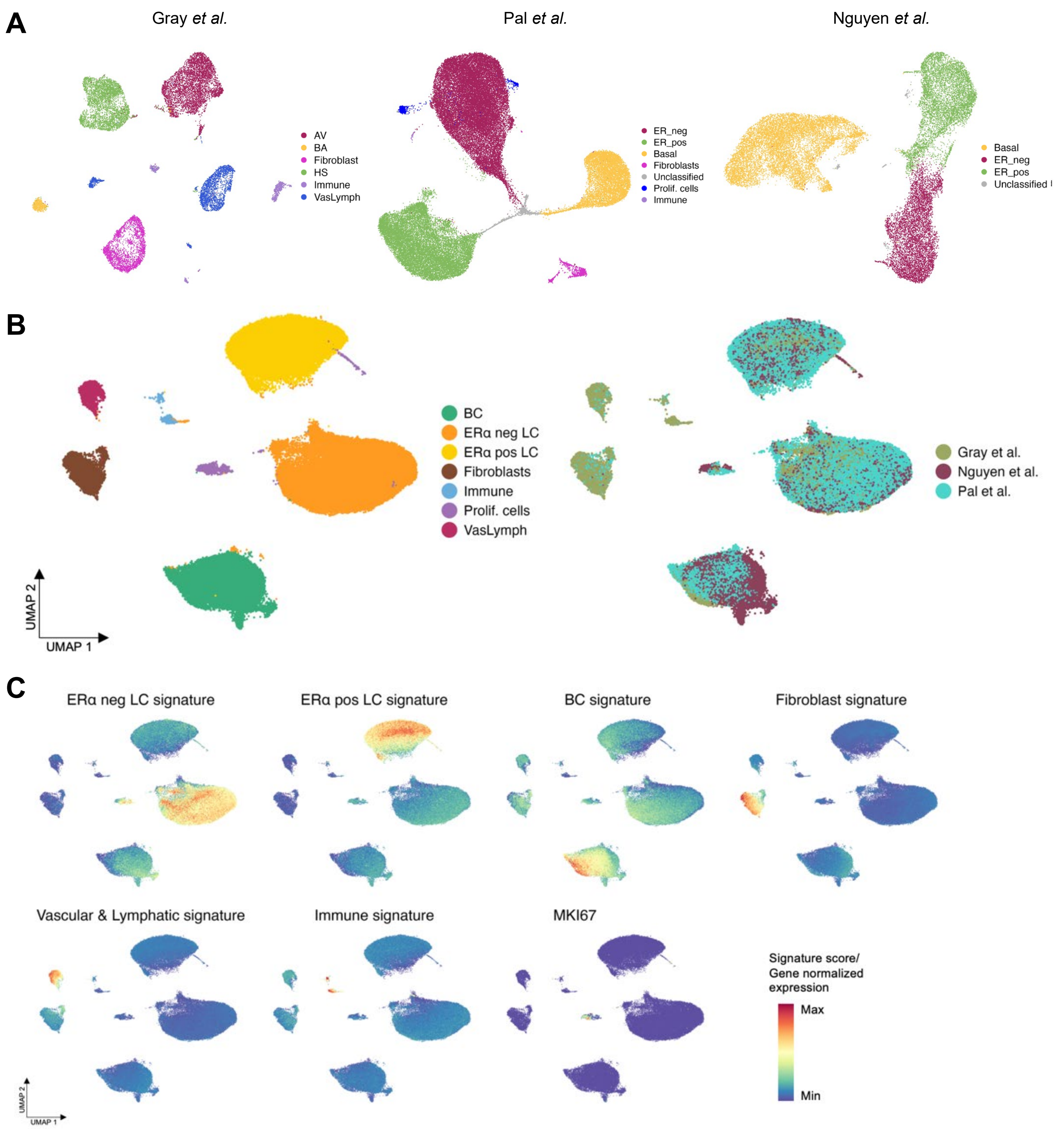

Supplementary Figure S4 | Integration of three scRNA-seq datasets from healthy human mammary gland. A, UMAP plots showing cell population distributions from three independent studies, labeled as Pal et al. [15], Gray et al. [21], and Nguyen et al. [14]. Each plot displays unique cell clusters with distinct expression profiles, categorized into cell types including immune cells, fibroblasts, and epithelial cells. **B**, UMAP plot of the integrated scRNA-seq human datasets, colored by cell type annotation (left), and by the dataset (right). **C,** UMAP plots showing the cell type-specific signature (Gray et al. [21]) scores for ERαneg LCs, ERαpos LCs, BaCs, fibroblasts, vascular and lymphatic and and immune cells.
