# Supplementary material for: Discovery and Evaluation of Biomarkers for Triple-Negative Breast Cancer Subtypes Uncovers Patient Stratification and Targeted Therapeutic Strategies
Source: Cancer Res. 2026 Feb 11;86(10):2360–76. doi: 10.1158/0008-5472.CAN-24-2758 (PMC13176827; doi:10.1158/0008-5472.CAN-24-2758)
Supplement: Supplementary Figure S5 — Immunohistochemical validation of basal markers in TNBC samples [file can-24-2758_supplementary_figure_s5_suppsf5.pdf]

Supplemental Figure 5

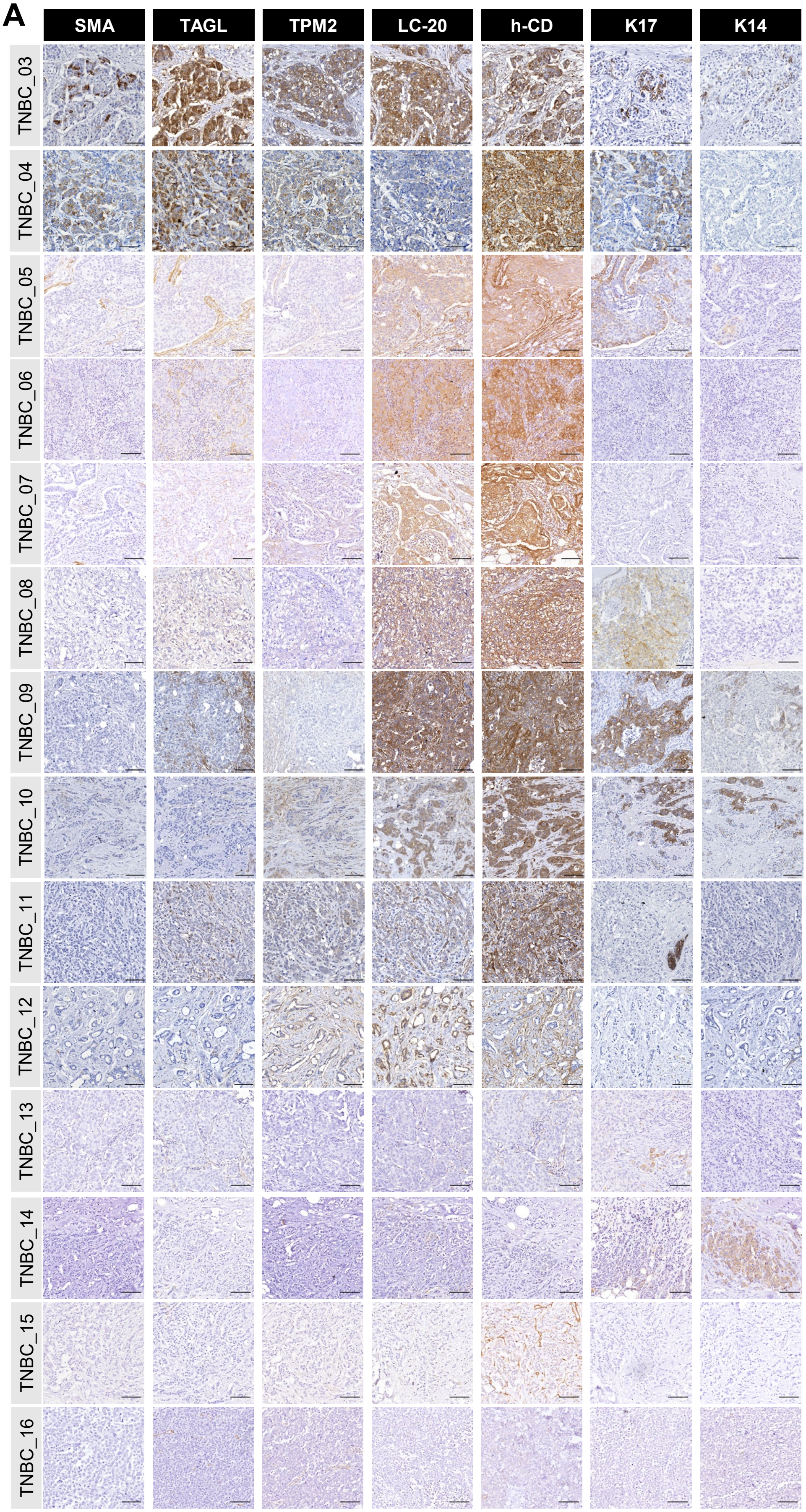

Supplemental Figure 5 (continued)

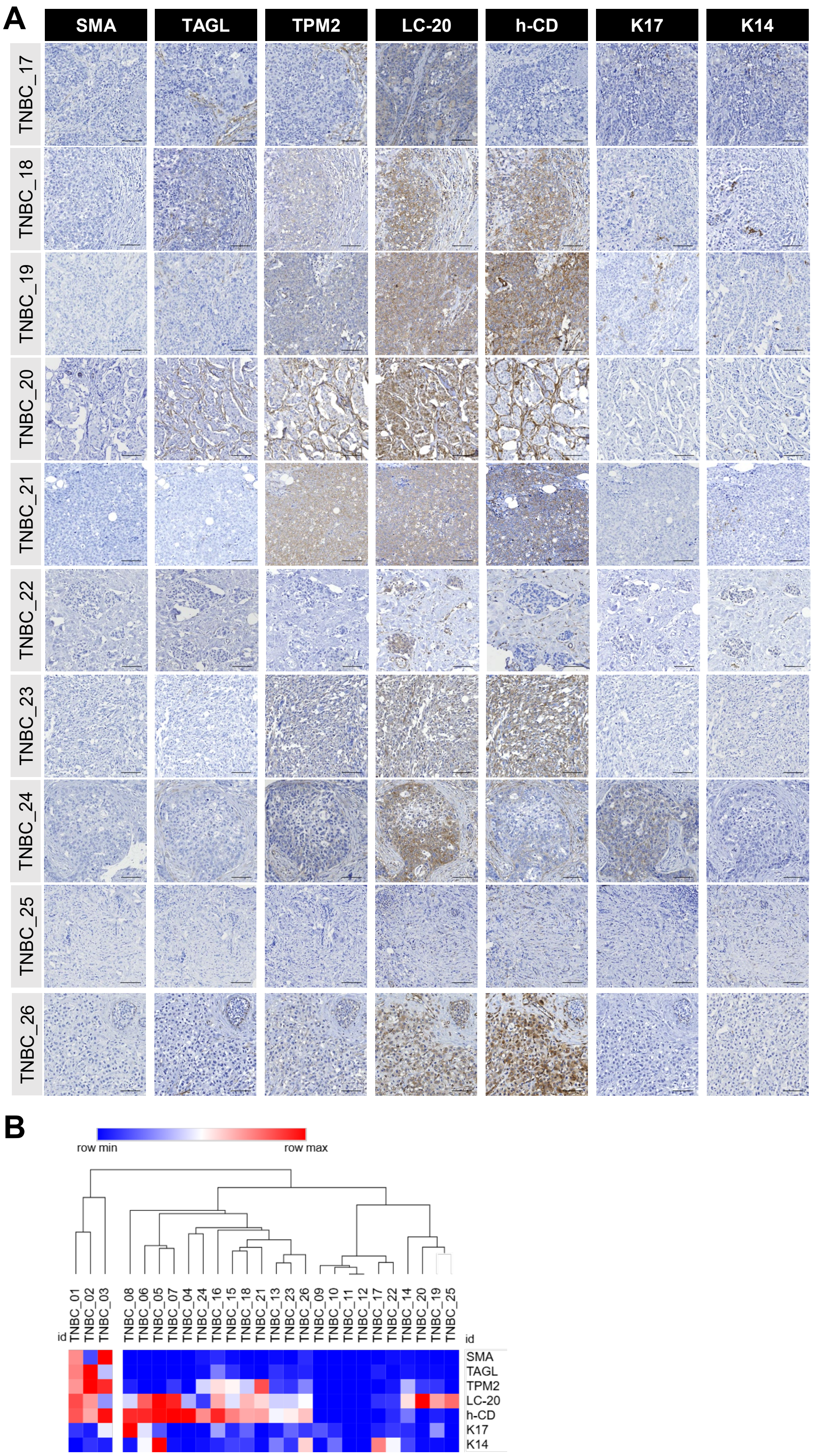

Supplemental Figure 5 | Immunohistochemical validation of basal markers in TNBC samples. A. Representative IHC staining of TNBC samples from the discovery cohort. Each row corresponds to a TNBC case, labeled from TNBC-02 to TNBC-26, and each column to a specific basal-associated marker (SMA, TAGL, TPM2, LC-20, h-CD, K17, and K14). Nuclei are counterstained with hematoxylin. Scale bar represents 100 µm. **B,** Quantitative heatmap summarizing marker expression across all TNBC samples in the discovery cohort based on the percentage of positive tumor cells. Red indicates high expression, blue indicates low/no expression, as determined by unsupervised clustering.
