# Supplementary material for: Discovery and Evaluation of Biomarkers for Triple-Negative Breast Cancer Subtypes Uncovers Patient Stratification and Targeted Therapeutic Strategies
Source: Cancer Res. 2026 Feb 11;86(10):2360–76. doi: 10.1158/0008-5472.CAN-24-2758 (PMC13176827; doi:10.1158/0008-5472.CAN-24-2758)
Supplement: Supplementary Figure S6 — Histological distribution of SMA, TAGL and TPM2 in the validation cohorts of TNBC [file can-24-2758_supplementary_figure_s6_suppsf6.pdf]

Supplementary Figure S6

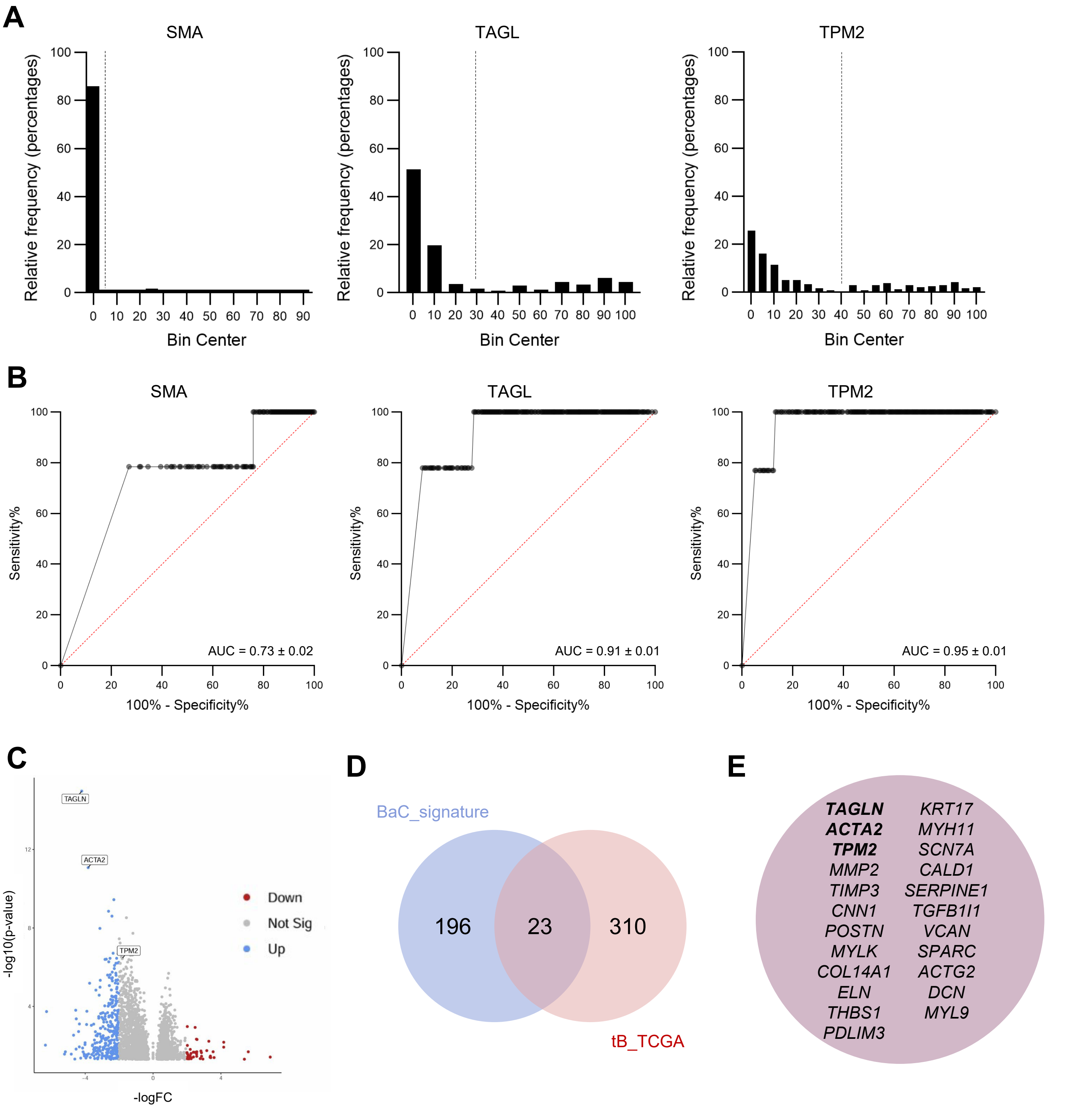

Supplementary Figure S6 | Histological distribution of SMA, TAGL and TPM2 in the validation cohorts of TNBC. A, Bar plots illustrating the relative frequency distribution of positive cells across the validation cohorts of TNBC for SMA, TAGL and TPM2. **B,** Receiver operating characteristic (ROC) curves for SMA (left), TAGL (middle), and TPM2 (right) showing the diagnostic performance of each tB-marker to discriminate tB-TNBC cases from nB-TNBC cases. The area under the curve (AUC) reflects the sensitivity and specificity of each marker, with high values indicating strong discriminatory power. **C,** Volcano plot showing the differential gene expression profile between tB-TNBC and nB-TNBC tumors based on bulk RNA-seq analysis [23]. Each point represents a gene; red indicates significantly downregulated, blue upregulated, and grey non-significant genes in nB-TNBC compared to tB-TNBC. Differentially expressed genes were identified using *DESeq2* with adjusted p-value < 0.05 and log2 fold-change > 1. **D,** Venn diagram comparing the set of genes upregulated in tB-TNBC tumors (tB-TCGA) with basal identity genes derived from single-cell analysis of normal mammary glands (BaC-signature). **E,** List of the 23 genes commonly shared between tB-TNBC and normal basal identity programs.
