# Supplementary material for: Discovery and Evaluation of Biomarkers for Triple-Negative Breast Cancer Subtypes Uncovers Patient Stratification and Targeted Therapeutic Strategies
Source: Cancer Res. 2026 Feb 11;86(10):2360–76. doi: 10.1158/0008-5472.CAN-24-2758 (PMC13176827; doi:10.1158/0008-5472.CAN-24-2758)
Supplement: Supplementary Figure S7 — Prognostic value of ACTA2, TAGLN, and TPM2 [file can-24-2758_supplementary_figure_s7_suppsf7.pdf]

Supplementary Figure S7

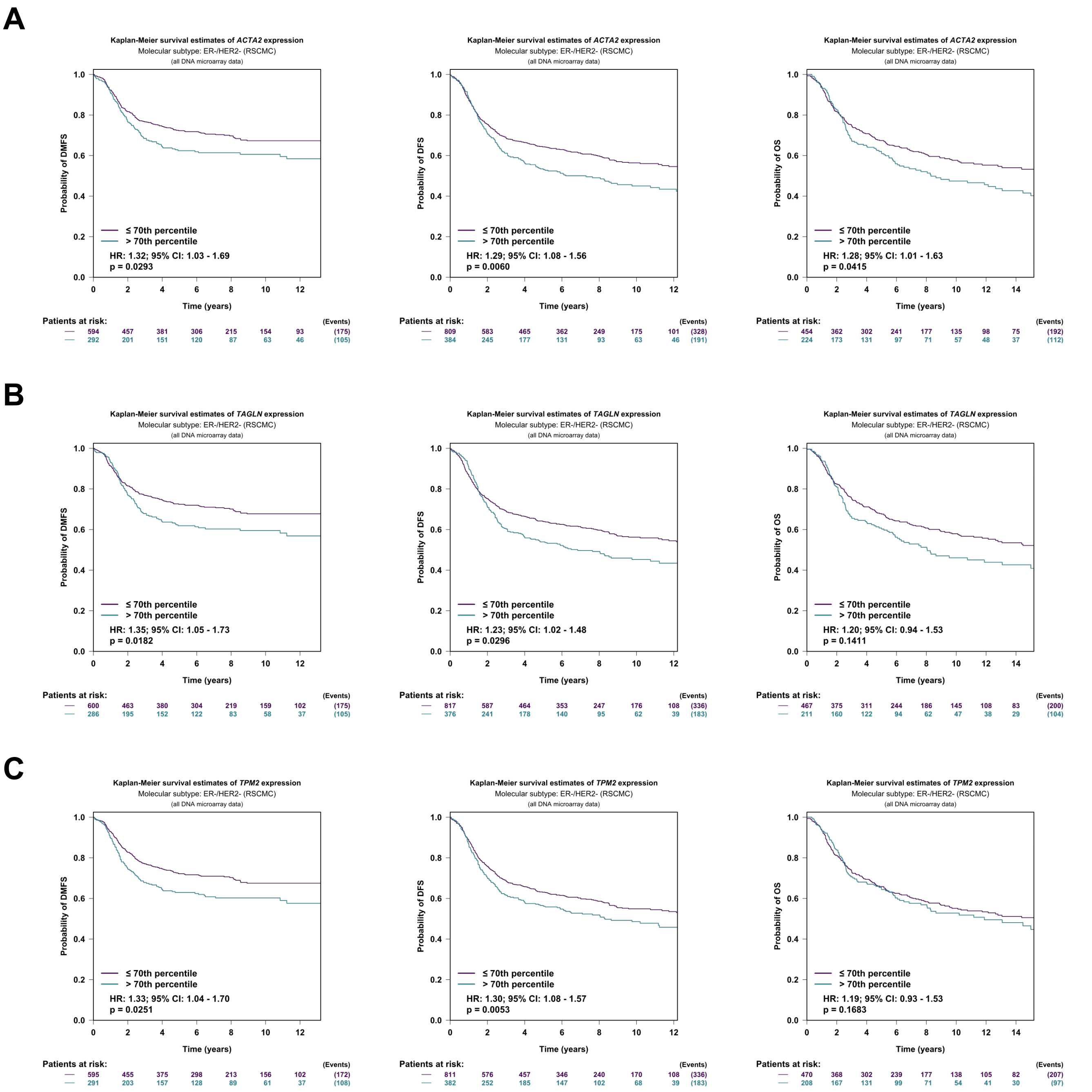

Supplementary Figure S7 | Prognostic value of *ACTA2, TAGLN*, and *TPM2*. KM plots illustrating the distant metastasis free survival (DMFS), disease-free survival (DFS) and overall survival (OS) of ER-/HER- (RSCMC) patients using *bc-GenExMiner* portal for *ACTA2* (**A**), *TAGLN* (**B**) and *TPM2* (**C**). Patient subgroups were defined comparing ≤70th percentile represented in purple versus >70th percentile shown in light blue. Hazard ratios (HR) with 95% confidence intervals (CIs) were calculated, along with P-values derived from the log-rank test. The number of patients at risk for mortality was reported beneath the main survival plot.
