# Supplementary material for: Discovery and Evaluation of Biomarkers for Triple-Negative Breast Cancer Subtypes Uncovers Patient Stratification and Targeted Therapeutic Strategies
Source: Cancer Res. 2026 Feb 11;86(10):2360–76. doi: 10.1158/0008-5472.CAN-24-2758 (PMC13176827; doi:10.1158/0008-5472.CAN-24-2758)
Supplement: Supplementary Figure S8 — Analysis of basal marker expression and immune checkpoint genes in TNBC [file can-24-2758_supplementary_figure_s8_suppsf8.pdf]

Supplementary Figure S8

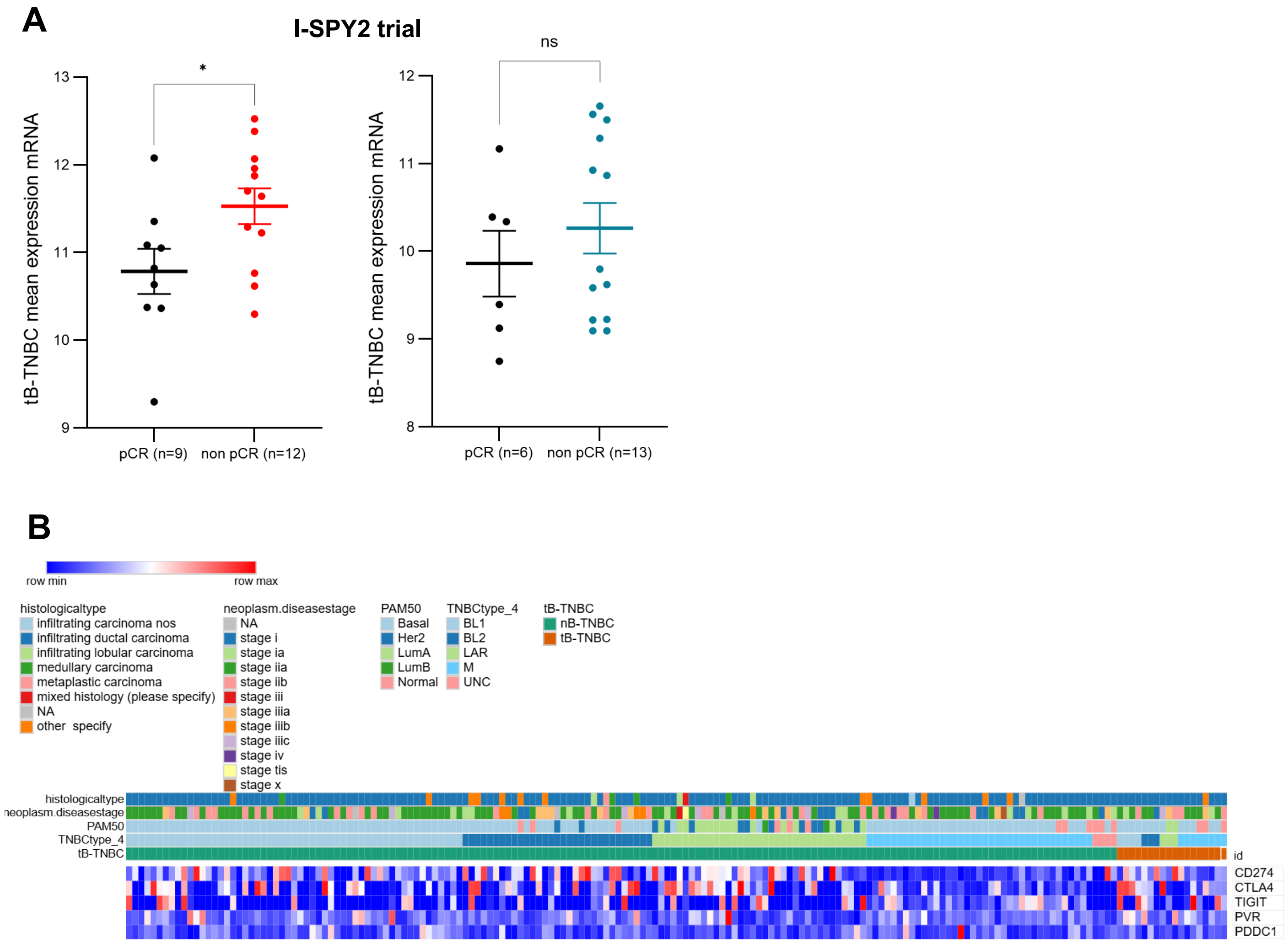

Supplementary Figure S8 | Analysis of basal marker expression and immune checkpoint genes in TNBC. A, Mean expression levels of basal markers (*TAGLN, ACTA2*, and *TPM2*) in TNBC patients treated in the I-SPY2 phase II clinical trial with durvalumab (anti-PD-L1), olaparib, and paclitaxel (left) and in TNBC control patients treated in the I-SPY2 phase II clinical trial treated with chemotherapy alone (taxol) (right). Patients are categorized as responders (pCR: complete pathological response, black dots) or non-responders (non-pCR, red dots or blue dots). Statistic was calculated using nonparametric t-test (*$p < 0.05$; mean ± SEM). **B**, Heatmap displaying the expression of immune checkpoint genes (*CD247, PDCD1, CTLA4, TIGIT*, and *PVR*) across TNBC samples. Rows represent individual immune checkpoint genes, and columns represent patient samples. Expression levels are color-coded from low (blue) to high (red). Annotations at the bottom indicate PAM50 subtypes (Basal, Her2, LumA, LumB, Normal), Lehmann subtypes (BL1, BL2, LAR, M, UNC), and tB-TNBC subclassification (orange: tB-TNBC; teal: nB-TNBC).
