# Supplementary material for: Discovery and Evaluation of Biomarkers for Triple-Negative Breast Cancer Subtypes Uncovers Patient Stratification and Targeted Therapeutic Strategies
Source: Cancer Res. 2026 Feb 11;86(10):2360–76. doi: 10.1158/0008-5472.CAN-24-2758 (PMC13176827; doi:10.1158/0008-5472.CAN-24-2758)
Supplement: Supplementary Figure S9 — Correlation between ACTA2, TAGLN, and TPM2 expression and drug sensitivity of selected compounds of drug screening [file can-24-2758_supplementary_figure_s9_suppsf9.pdf]

Supplementary Figure S9

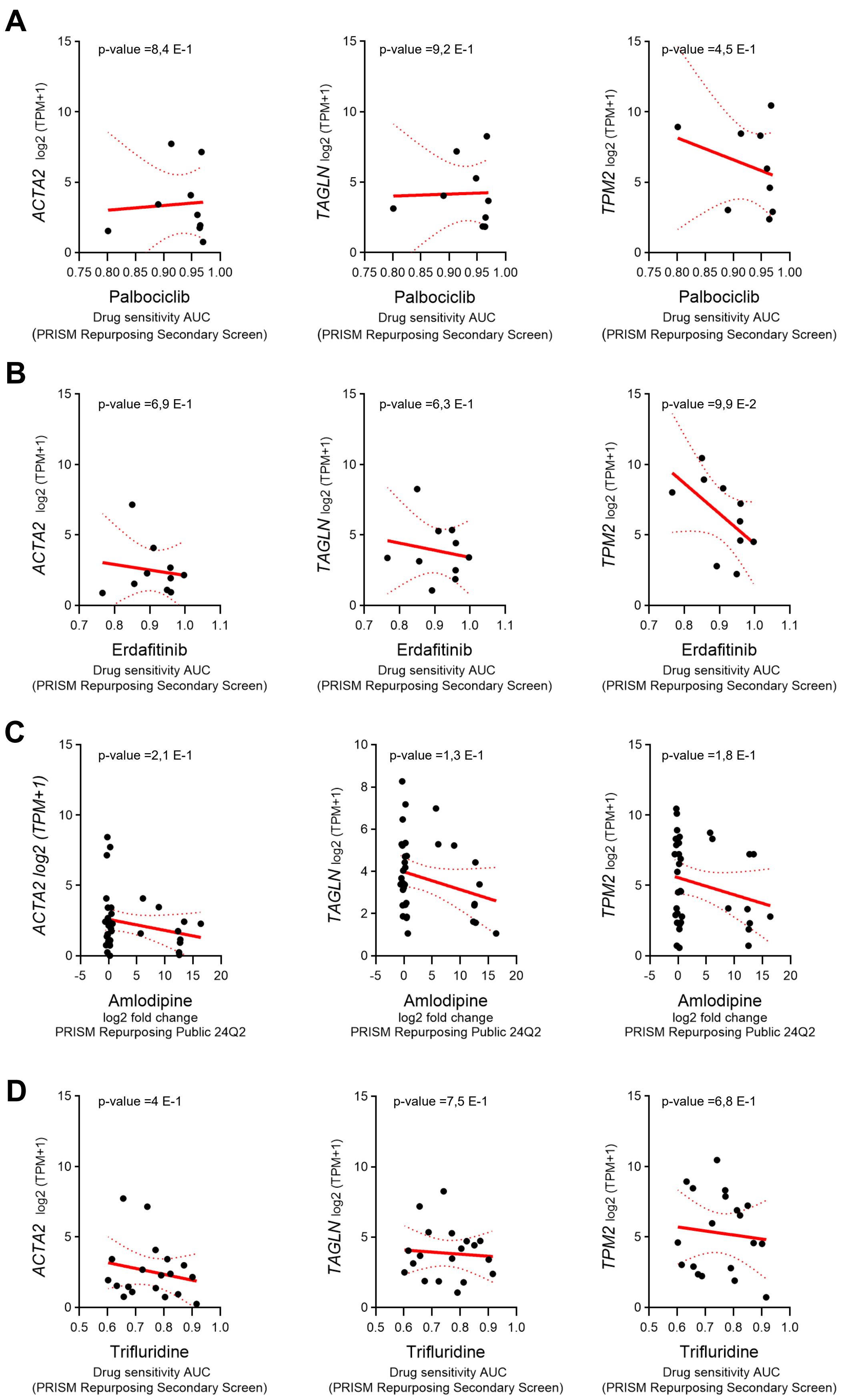

Supplementary Figure S9 | Correlation between *ACTA2*, *TAGLN*, and *TPM2* expression and drug sensitivity of selected compounds of drug screening. Correlation plots showing the association between *ACTA2*, *TAGLN*, and *TPM2* expression (log2 TPM+1) and palbociclib sensitivity (AUC) (**A**), Erdafitinib sensitivity (AUC) (**B**), Amlodipine sensitivity (log2 fold change) (**C**) and Trifluridine sensitivity (AUC) (**D**) in breast cancer cell lines. Red lines indicate regression fit; shaded areas show 95% confidence intervals. P-values calculated by Pearson correlation.
