# Supplementary material for: Discovery and Evaluation of Biomarkers for Triple-Negative Breast Cancer Subtypes Uncovers Patient Stratification and Targeted Therapeutic Strategies
Source: Cancer Res. 2026 Feb 11;86(10):2360–76. doi: 10.1158/0008-5472.CAN-24-2758 (PMC13176827; doi:10.1158/0008-5472.CAN-24-2758)
Supplement: Supplementary Figure S10 — Functional validation of TAGLN-KO cells [file can-24-2758_supplementary_figure_s10_suppsf10.pdf]

Supplementary Figure S10

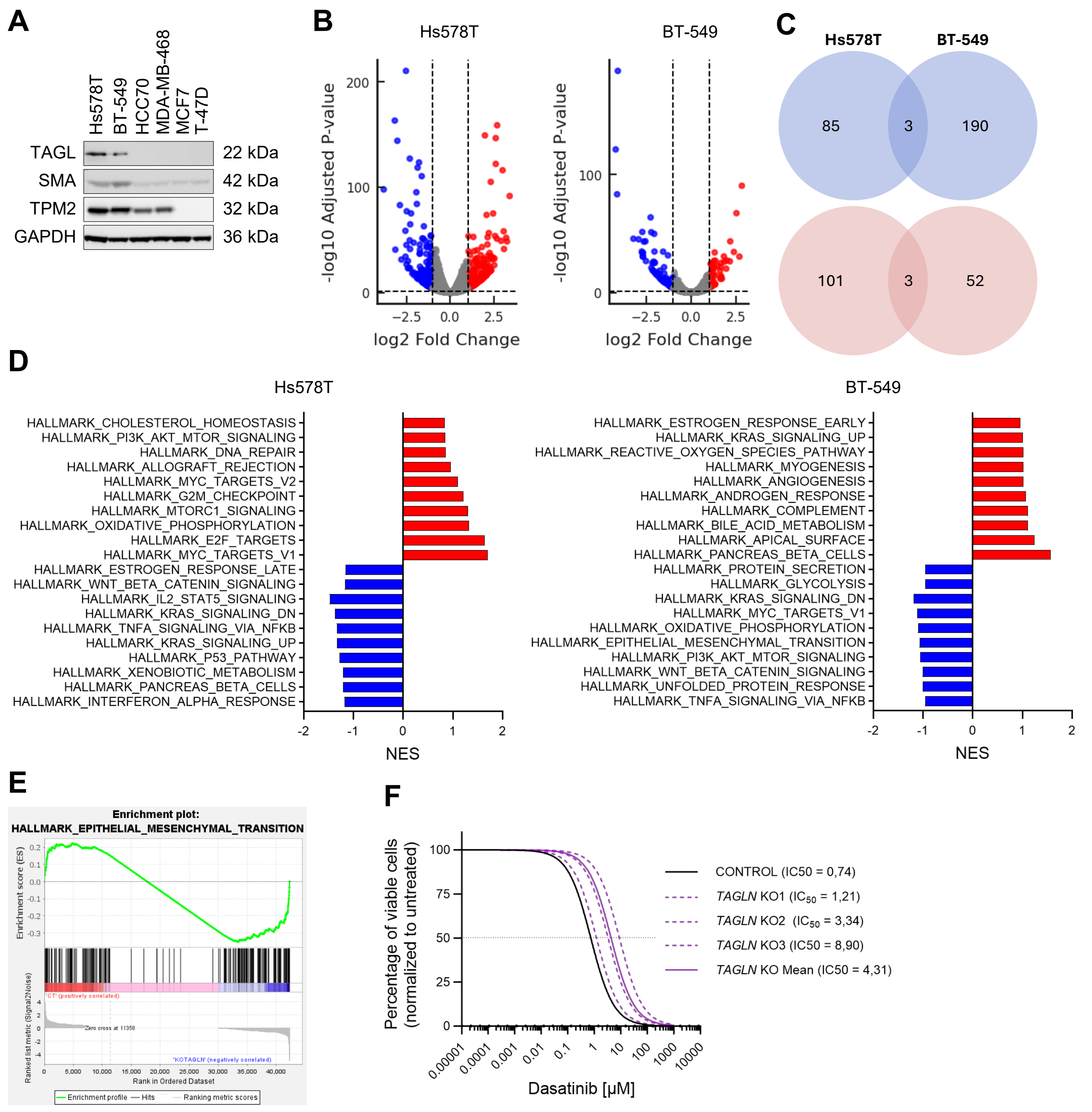

Supplementary Figure S10 | Functional validation of TAGLN-KO cells. A, WB analysis comparing the expression of tB-markers in the screened breast cancer cell lines. **B,** Volcano plots showing differentially expressed genes in *TAGLN*-KO cells compared to CONTROL Hs578T and BT-549 cells. **C,** Venn diagrams summarizing the overlap of DEGs between Hs578T and BT-549 cells. Top: downregulated genes; bottom: upregulated genes. **D,** Bar graph representing the normalized enrichment score (NES) of gene sets in *TAGLN*-KO compared to CONTROL Hs578T and BT-549 cells (positive NES in red, negative NES in blue). **E,** Enrichment plot showing the GSEA for EMT of *TAGLN*-KO compared to CONTROL BT-549 cells. **F,** Drug-response curves for cell viability of BT-549 cells, comparing CONTROL (black) and TAGLN-KO (purple) treated with increasing concentrations of dasatinib. Solid lines represent the mean of three biological replicates, performed in technical replicates. Dashed lines indicate the IC_{50} for each condition. Statistical significance for differential expression was calculated using DESeq2; gene set enrichment was evaluated using GSEA with FDR q-value < 0.25 as significance threshold.
