# Supplementary material for: Discovery and Evaluation of Biomarkers for Triple-Negative Breast Cancer Subtypes Uncovers Patient Stratification and Targeted Therapeutic Strategies
Source: Cancer Res. 2026 Feb 11;86(10):2360–76. doi: 10.1158/0008-5472.CAN-24-2758 (PMC13176827; doi:10.1158/0008-5472.CAN-24-2758)
Supplement: Supplementary Figure S11 — Analysis of TAGLN expression and dasatinib target interaction in cancer cell lines [file can-24-2758_supplementary_figure_s11_suppsf11.pdf]

Supplementary Figure S11

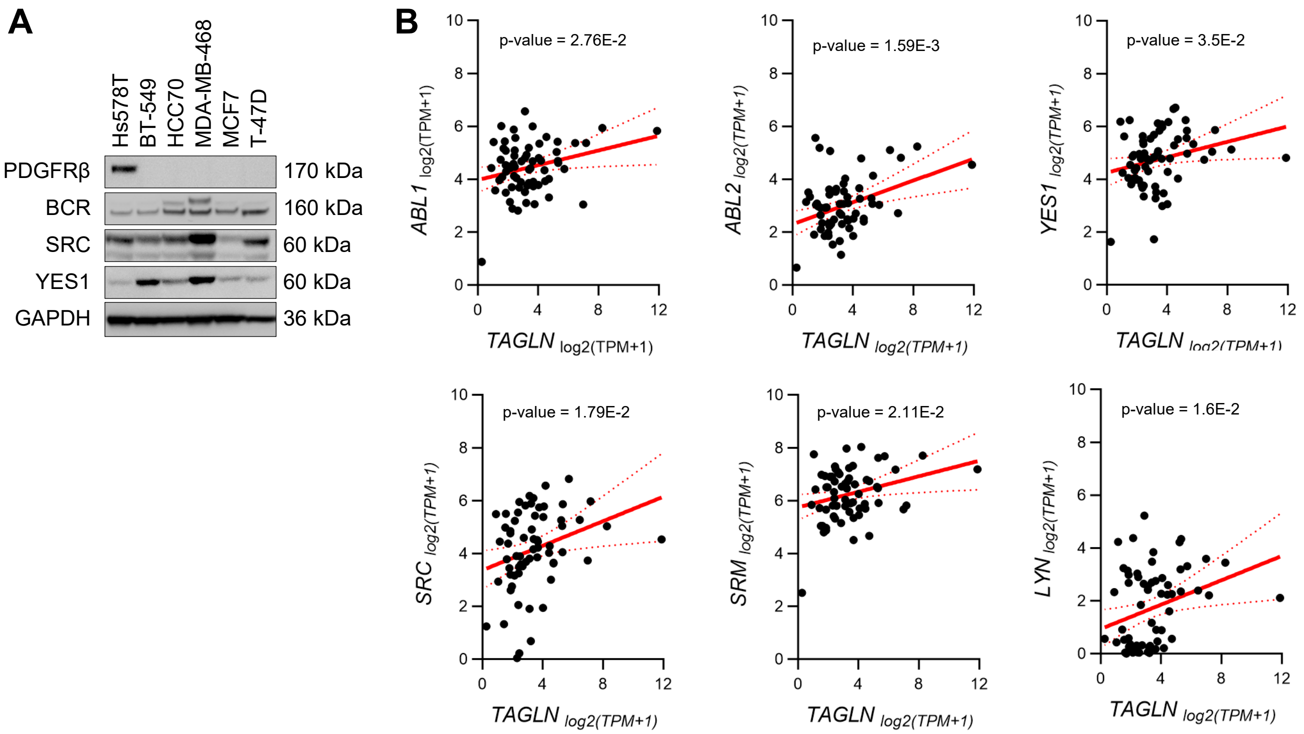

Supplementary Figure S11 | Analysis of *TAGLN* expression and dasatinib target interaction in cancer cell lines. A, WB analysis showing protein levels of TAGL and indicated dasatinib targets in breast cancer cell lines. **B,** Scatter plots displaying the correlation between *TAGLN* expression and the expression of dasatinib target genes (*ABL1, ABL2, YES1, LYN, SRC*, and *SRM*) in breast cancer cell lines. Each plot shows the expression of *TAGLN* (log2(TPM+1)) on the X-axis against the expression of a target gene (log2(TPM+1)) on the Y-axis. Data points are depicted as black dots, with red dashed lines indicating the trend lines and p-values provided for each correlation.
