# Supplementary material for: Discovery and Evaluation of Biomarkers for Triple-Negative Breast Cancer Subtypes Uncovers Patient Stratification and Targeted Therapeutic Strategies
Source: Cancer Res. 2026 Feb 11;86(10):2360–76. doi: 10.1158/0008-5472.CAN-24-2758 (PMC13176827; doi:10.1158/0008-5472.CAN-24-2758)
Supplement: Supplementary Table S1 — Distribution of clinical and pathological features in the validation cohort BR1301a [file can-24-2758_supplementary_table_s1_suppst1.pdf]

Supplementary Table S1

Variable	tB-TNBC mean ± SEM and/or distribution [N=17]	nB-TNBC mean ± SEM and/or distribution [N=103]	Total
**Demographics**			
Age	51,06 ± 2,486 (13,9%)	49,91 ± 1,061 (86%)	120
**TNM Classification**			
**Tumor size**			
T1	3 (17,65%)	7 (6,8%)	10
T2	10 (58,82%)	72 (69,90%)	82
T3	4 (23,5%)	19 (18,45%)	23
T4	0 (0%)	5 (4,85%)	5
**Nodal status**			
N0	14 (82,35%)	66 (64,08%)	80
N1	2 (11,76%)	20 (19,42%)	22
N2	1 (5,88%)	11 (10,68%)	12
N3	0 (0%)	6 (5,82%)	6
**Metastasis**			
M0	17 (100%)	103 (100%)	120
M1	0 (0%)	0 (0%)	0
**Tumor grade**			
Grade 1	0 (0%)	2 (1,68%)	2
Grade 2	8 (47,1%)	33 (32,67%)	41
Grade 3	9 (52,9%)	66 (63,35%)	75
**Clinical Stage**			
I	3 (17,65%)	3 (2,91%)	6
II	13 (76,47%)	79 (76,70%)	92
III	1 (5,88%)	21 (20,39%)	22
IV	0 (0%)	0 (0%)	0
**Molecular markers**			
SMA****	48,18 ± 7,124	1,03 ± 0,395	120
TAGL****	50,18 ± 8,062	2,937± 0,327	120
TPM2****	65,66 ± 6,812	10,41 ± 2,086	120

Table S1 | Distribution of clinical and pathological features in the validation cohort BR1301a. This table summarizes the clinical-pathological characteristics and the expression of molecular markers of TNBC patients, classified into tB and nB-TNBC based on the staining for SMA, TAGL, and TPM2. The TNM classification is detailed along with tumor grade and clinical stage. Each category is reported with the number of patients (n) and the percentage (%) relative to the total number of patients in the respective group. Age and Molecular markers mean percentage expression ± SEM is represented for each group. The total column reflects the overall count for each category. Statistical significance is denoted by asterisk (****, $p<0.0001$). Clinical-pathological characteristics statistical analysis was performed using Fisher's exact test. Age statistical analysis was performed using nonparametric *t-Test*. Molecular markers statistical analysis was carried out using two-way ANOVA.
