# Supplementary material for: Discovery and Evaluation of Biomarkers for Triple-Negative Breast Cancer Subtypes Uncovers Patient Stratification and Targeted Therapeutic Strategies
Source: Cancer Res. 2026 Feb 11;86(10):2360–76. doi: 10.1158/0008-5472.CAN-24-2758 (PMC13176827; doi:10.1158/0008-5472.CAN-24-2758)
Supplement: Supplementary Table S2 — Distribution of clinical and pathological features in the validation cohort of Hospital Universitario Santa Lucía, Cartagena [file can-24-2758_supplementary_table_s2_suppst2.pdf]

Supplementary Table S2

Variable	tB-TNBC mean ± SEM and/or distribution [N=38]	nB-TNBC mean ± SEM and/or distribution [N=85]	Total
**Demographics**			
Age*	63.57± 2.194	57.85 ± 1.658	123
**TNM Classification**			
**Tumor size***			
T1	18/36 (50%)	21/78 (26.92%)	39
T2	9/36 (25%)	40/78 (51.28%)	49
T3	6/36 (16.67%)	11/78 (14.1%)	17
T4	3/36 (8.33%)	6/78 (7.69%)	9
**Nodal status**			
N0	20/33 (60.61%)	37/73 (50.68%)	57
N1	10/33 (30.3%)	22/73 (30.14%)	32
N2	2/33 (6.06%)	7/73 (9.59%)	9
N3	1/33 (3.03%)	7/73 (9.59%)	8
**Metastasis**			
M0	30/37 (81.08%)	61/86 (70.93%)	91
M1	7/37 (18.92%)	25/86 (29.07%)	32
**Tumor grade**			
Grade 1	1/32 (3.13%)	0/84 (0%)	1
Grade 2	22/32 (68.75%)	16/84 (19.05%)	38
Grade 3	9/32 (28.13%)	68/84 (80.95%)	77
**Histological subtype**			
NOS	29/37 (78.38%)	69/85 (81.18%)	98
Medullary	2/37 (5.41%)	11/85 (12.94%)	13
Apocrine	3/37 (8.11%)	2/85 (2.35%)	5
Metaplastic	1/37 (2.7%)	1/85 (1.18%)	2
Lobular	0/37 (0%)	1/85 (1.18%)	1
Squamous	0/37 (0%)	1/85 (1.18%)	1
Adenoid Cystic Carcinoma	2/37 (5.41%)	0/85 (0%)	2
**Molecular markers**			
SMA*	9.48 ± 3.198	0.899 ± 0.537	123
TAGL****	77.82 ± 3.628	14.38± 2.629	123
TPM2****	73.77 ± 2.974	22.66 ± 3.141	123

Table S2 | Distribution of clinical and pathological features in the validation cohort of Hospital Universitario Santa Lucía, Cartagena. This table illustrates the clinical-pathological characteristics of TNBC patients, classified into tB and nB-TNBC based on the staining for SMA, TAGL, and TPM2. The TNM classification is detailed along with tumor grade, and histological subtype. Each category is reported with the number of patients (n) and the percentage (%) relative to the total number of patients in the respective group. Mean age ± SEM is represented for each group. The total column reflects the overall count for each category. Statistical significance is denoted by asterisk (****, p<0,0001; *, p-value <0,05). Clinical-pathological characteristics statistical analysis was performed using Fisher's exact test. Age statistical analysis was performed using nonparametric *t-test*. Molecular markers statistical analysis was carried out using two-way ANOVA. Not otherwise specified (NOS).
