# Supplementary material for: Discovery and Evaluation of Biomarkers for Triple-Negative Breast Cancer Subtypes Uncovers Patient Stratification and Targeted Therapeutic Strategies
Source: Cancer Res. 2026 Feb 11;86(10):2360–76. doi: 10.1158/0008-5472.CAN-24-2758 (PMC13176827; doi:10.1158/0008-5472.CAN-24-2758)
Supplement: Supplementary Table S3 — Primary antibodies used for immunohistochemistry [file can-24-2758_supplementary_table_s3_suppst3.pdf]

Supplementary Table S3

Target	Reference	RRID	Supplier	[IHC]	Positive control	Staining
Smooth Muscle Actin Anticuerpo (B4)	sc-53142	AB_2273670	Santa cruz	2 ug/ml	Smooth muscle	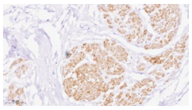
Transgelin Anticuerpo (6G6)	sc-53932	AB_1129519	Santa cruz	2 ug/ml	Smooth muscle	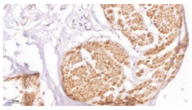
CXCL14 Polyclonal Antibody	PA5-106402	AB_2854075	Invitrogen	2 ug/ml	Adrenal gland	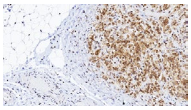
Tropomyosin Anticuerpo (F-6)	sc-74480	AB_2272017	Santa cruz	1 ug/ml	Smooth muscle	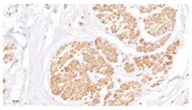
Anti-KRT14 antibody	HPA023040	AB_1852201	Sigma-Aldrich	0,1 mg/ml	Skin	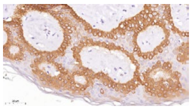
SPARC Anticuerpo (AON-1)	sc-33645	AB_628057	Santa cruz	2 ug/ml	Kidney	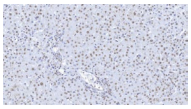
Anti-CALD1 antibody	HPA017330	AB_1845917	Sigma-Aldrich	0,7 mg/ml	Smooth muscle	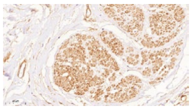
MYL9/MYL12A/B Anticuerpo (E-4)	sc-28329	AB_2282358	Santa cruz	1 ug/ml	Smooth muscle	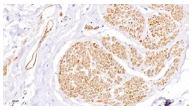
Cytokeratin 17 Anticuerpo (E-4)	sc-393002	AB_2893006	Santa cruz	2 ug/ml	Skin	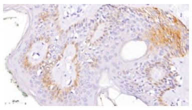
Apolipoprotein E/apoE Anticuerpo (WU E-4)	sc-53570	AB_781458	Santa cruz	2 ug/ml	Adrenal gland	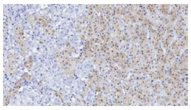

Table S3 | Primary antibodies used for immunohistochemistry. This table lists the antibodies utilized across various assays in the study. Details include the target protein, the catalog number, the RRID code, supplier, the specific concentrations used for IHC, the positive control tissue and representative staining of positive control. Concentrations are provided in micrograms per milliliter (µg/ml) or milligrams per milliliter (mg/ml) as appropriate.
