# Supplementary material for: Discovery and Evaluation of Biomarkers for Triple-Negative Breast Cancer Subtypes Uncovers Patient Stratification and Targeted Therapeutic Strategies
Source: Cancer Res. 2026 Feb 11;86(10):2360–76. doi: 10.1158/0008-5472.CAN-24-2758 (PMC13176827; doi:10.1158/0008-5472.CAN-24-2758)
Supplement: Supplementary Table S4 — Secondary antibodies used for IHC, IF, and WB assays [file can-24-2758_supplementary_table_s4_suppst4.pdf]

Supplementary Table S4

Target	Reference	RRID	Supplier	[IHC]	[IF]	[WB]
Goat anti-Mouse IgG (H+L) Secondary Antibody, HRP	31430	AB_228307	Thermo Scientific	3,2 ug/ml	-	0,1 ug/ml
Goat anti-Rabbit IgG (H+L) Secondary Antibody, HRP	31460	AB_228341	Thermo Scientific	3,2 ug/ml	-	0,1 ug/ml
Alexa Fluor® 488 AffiniPure Donkey Anti-Mouse IgG (H+L)	715-545-150	AB_2340846	Jackson Immunoresearch	-	1 ug/ml	

Table S4 | Secondary antibodies used for immunohistochemistry (IHC), immunofluorescence (IF), and Western blot (WB) assays. This table lists the antibodies utilized across various assays in the study. Details include the target protein, the catalog number, the RRID code, supplier, and the specific concentrations used for each assay type (IHC, IF, WB). Concentrations are provided in micrograms per milliliter (µg/ml) or milligrams per milliliter (mg/ml) as appropriate. Antibodies are grouped by their respective applications, providing a comprehensive resource for replication of the experimental conditions.
