# Supplementary material for: Discovery and Evaluation of Biomarkers for Triple-Negative Breast Cancer Subtypes Uncovers Patient Stratification and Targeted Therapeutic Strategies
Source: Cancer Res. 2026 Feb 11;86(10):2360–76. doi: 10.1158/0008-5472.CAN-24-2758 (PMC13176827; doi:10.1158/0008-5472.CAN-24-2758)
Supplement: Supplementary Table S5 — Primary antibodies used in IF and WB assays [file can-24-2758_supplementary_table_s5_suppst5.pdf]

Supplementary Table S5

Target	Reference	RRID	Supplier	[IF]	[WB]
Transgelin Antibody (6G6)	sc-53932	AB_1129519	Santa cruz	2 ug/ml	0,2 ug/ml
BCR Polyclonal Antibody	PA5-17709	AB_10982586	Invitrogen		0,45 ug/ml
Rabbit anti-PDGFR beta Recombinant Monoclonal Antibody [BLR081G]	A700-081-T	AB_2891878	Bethyl Laboratories		1 ug/ml
SRC Monoclonal Antibody (184Q20)	AHO1152	AB_1500518	Invitrogen		0,5 ug/ml
GAPDH Antibody (0411)	sc-47724	AB_627678	Santa cruz		0,2 ug/ml
YES1-Specific Polyclonal antibody	20243-1-AP	AB_10697656	Proteintech		1,6 ug/ml
ABL1/ABL2 Poycloncal antibody	PA5-114805	AB_2899441	INVITROGEN		1 ug/ml

Table S5 | Primary antibodies used immunofluorescence (IF), and Western blot (WB) assays. This table lists the antibodies utilized across various assays in the study. Details include the target protein, the catalog number, the RRID code, supplier, and the specific concentrations used for each assay type (IF and WB). Concentrations are provided in micrograms per milliliter (µg/ml) or milligrams per milliliter (mg/ml) as appropriate. Antibodies are grouped by their respective applications, providing a comprehensive resource for replication of the experimental conditions.
